# Near-infrared-II ratiometric fluorescence probes for non-invasive detection and precise navigation surgery of metastatic sentinel lymph nodes

**DOI:** 10.7150/thno.78085

**Published:** 2022-10-09

**Authors:** Mengfei Li, Xue Zheng, Tianyang Han, Shengjie Ma, Yajun Wang, Bin Sun, Jiajun Xu, Xin Wang, Songling Zhang, Shoujun Zhu, Xiaoyuan Chen

**Affiliations:** 1 Joint Laboratory of Opto-Functional Theranostics in Medicine and Chemistry, The First Hospital of Jilin University, Changchun 130021, China.; 2 State Key Laboratory of Supramolecular Structure and Materials, College of Chemistry, Jilin University, Changchun 130012, China.; 3 Department of Gastrointestinal Surgery, The First Hospital of Jilin University, Changchun 130021, China.; 4 Department of Obstetrics and Gynecology, The First Hospital of Jilin University, Changchun, 130021, China.; 5 Departments of Diagnostic Radiology, Surgery, Chemical and Biomolecular Engineering, and Biomedical Engineering, Yong Loo Lin School of Medicine and College of Design and Engineering, National University of Singapore, Singapore 119074, Singapore.; 6 Clinical Imaging Research Centre, Centre for Translational Medicine, Yong Loo Lin School of Medicine, National University of Singapore, Singapore 117599, Singapore.; 7 Nanomedicine Translational Research Program, NUS Center for Nanomedicine, Yong Loo Lin School of Medicine, National University of Singapore, Singapore 117597, Singapore.

**Keywords:** ratiometric fluorescence, non-invasive detection, identification of tumor-draining lymph nodes, sentinel lymph node, near-infrared-II imaging-guided surgery

## Abstract

Sentinel lymph node (SLN) biopsy is the key diagnostic procedure to determine tumor metastasis and treatment plan. Current SLN biopsy has considerable drawbacks in that SLNs (both malignant and normal) must be removed by navigation surgery, followed by a time-consuming pathological examination. The selective, non-invasive, and real-time diagnosis of metastatic status in SLNs is becoming essential.

**Methods:** Here, we design two lanthanide-doped nanoparticles as a pair of NIR-II ratiometric fluorescence probes, one of which is conjugated with tumor-targeting moiety, while the other is conjugated with PEG as an internal reference. The NIR-II ratiometric fluorescence signal (I_1060 nm_/I_1525 nm_) from two well-separated channels were used to identify the tumor-draining SLNs. The precise navigation surgery of metastatic SLNs was performed and we further evaluated their surgery outcomes.

**Results:** The NIR-II ratiometric fluorescence facilitates an ideal fluorescence-guided surgery with only resection of tumor-positive SLNs, thereby avoiding unnecessary removal of the normal SLNs. In addition, our system has a time-saving operation procedure and can be performed under the operation light without altering the appearance of surgical settings.

**Conclusion:** The present study enables non-invasive and real-time detection metastatic status in SLNs with high sensitivity and selectivity. Our investigations will provide a new direction for SLN biopsy and substantially improve cancer surgery outcomes.

## Introduction

The sentinel lymph nodes (SLNs) are considered as the first station of tumor metastasis 1-3. SLN biopsy has been recommended as a common operation to determine tumor staging, treatment decision-making, and prognosis-planning for a wide variety of cancers, such as breast cancer, cervical cancer, and melanoma 4, 5. The standard SLN biopsy includes intraoperative lymphangiography with contrast agent and subsequent pathological assessment 6. However, current SLN mapping in clinic relies on the injection of a radioactive tracer for visualization of the SLNs, which suffers from radiation exposure and low spatiotemporal resolution 7, 8. Alternative near-infrared (NIR) fluorescence imaging is inherently safe and has benefited the field of fluorescence-guided surgery (FGS) 9-12. However, clinically-available contrast agents are limited by photobleaching issue and low imaging contrast, let alone the *in vivo* identification of tumor-positive SLNs 13-15. The required pathological assessment for lesion identification in clinic also has considerable shortcomings, given that the intraoperative frozen section lacks sufficient accuracy while the paraffin section requires time-consuming procedures 16. In addition, pathological identification can only be operated by a skilled pathologist to provide accurate outputs. As SLNs dissection may cause morbidity of lymphedema, neurovascular injury, dysfunction, etc*.* 17, 18, it is thus essential to develop an effective and accurate approach to identify and excise tumor-infiltrating SLNs.

Many efforts have been made to determine the metastatic SLNs by using MRI nanoparticles 19-21, surface-enhanced Raman nanotags 22, 23, ultrasound microbubbles 24-26, and photoacoustic probes 27-29 to label SLNs with tumor-targeting function. Nevertheless, the visualization strategies using single signal output generally cause considerable false-positive results due to the off-target binding, the pressure in the tumor, and variations of permeability and retention in SLNs 30-32. In addition, this disadvantage is further aggravated when the output signals are inherently low for some of the applied imaging modalities. Recently, the ratiometric strategy, which relies on a self-calibration of signal intensity rather than single signal output, is promising to achieve quantitative targeting imaging 33-35. Therefore, ratiometric Raman nanotags were reported to facilitate more accurate and reliable SLNs pathologic evaluation 23, yet they failed to real-time collect adequate data from living samples.

Despite the excitement of NIR FGS for surgical resection of tumors, NIR-II (900-3000 nm) contrast agents have been shown to be superior to clinical NIR-I imaging agents with improved penetration depth and imaging contrast 36-43. Several influential studies have applied ratiometric strategy for *in vivo* detection of various analyte such as ClO^-^, ONOO^-^, and GSH 44-48. In these studies, NIR-II ratiometric fluorescence was recorded from two analyte-induced signal fluctuations, in which one signal was regarded as a reference to normalize the detective signal, and therefore ratiometric fluorescence imaging was independent of agent's concentration and interference factors. Given these backgrounds, employing ratiometric fluorescence imaging is likely promising to achieve an ideal intraoperative metastatic SLNs biopsy.

**Scheme 1:**
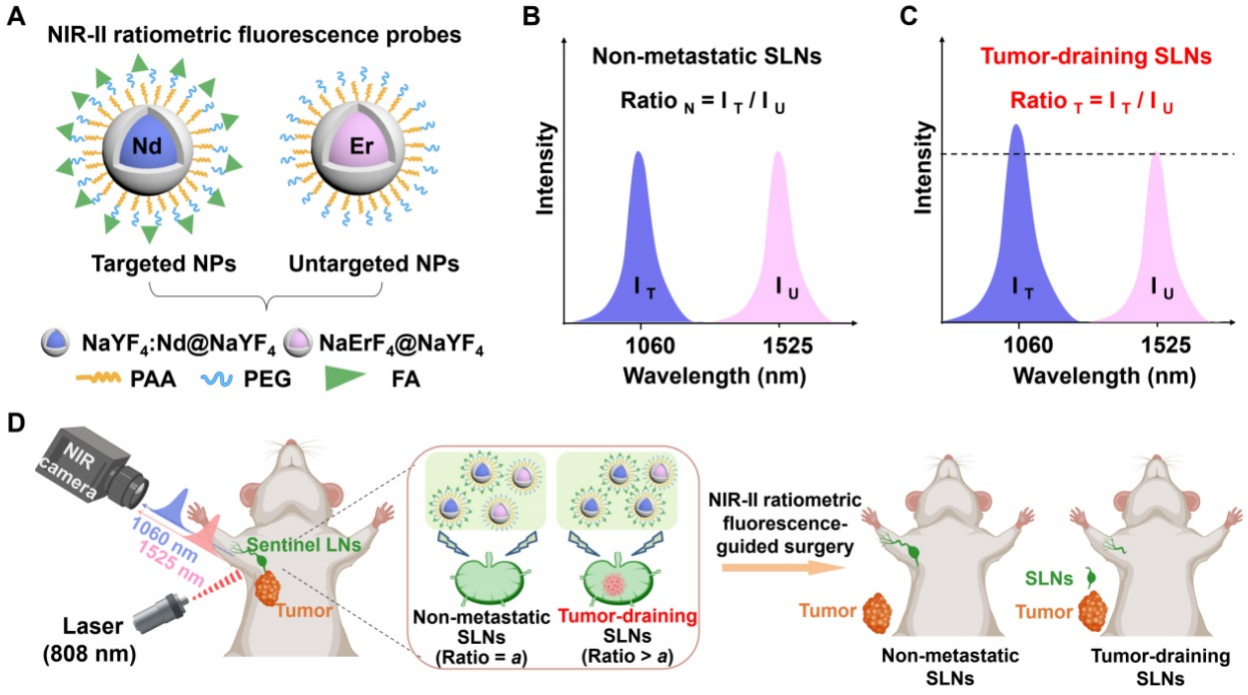
Schematic illustration of NIR-II ratiometric fluorescence probes for real-time non-invasive detection of tumor-draining lymph nodes. **(A)** Schematic diagrams of NIR-II ratiometric fluorescence probes. **(B, C)** Detection principle of NIR-II ratiometric fluorescence probes with non-overlapping wavelength emission. **(D)** NIR-II ratiometric fluorescence probes for real-time non-invasive detection of tumor-draining lymph nodes. Images were created with BioRender.com.

Towards this goal, we developed a set of NIR-II ratiometric fluorescence probes for non-invasive identification of metastatic SLNs in breast cancer. As depicted in **Scheme 1A**, the ratiometric fluorescence probes were composed of neodymium (Nd)-doped nanoparticles (Nd@Y NPs) with 1060 nm emission and erbium (Er)-doped nanoparticles (Er@Y NPs) with 1525 nm emission. The Nd@Y NPs were functionalized with folic acid (FA), which can selectively bind to the folate receptor of various cancers 49, 50, thus serving as the tumor-targeted probe (Nd@Y-FA). Concurrently, Er@Y NPs were modified with polyethylene glycol (PEG) as a non-targeted probe (Er@Y-PEG) and an internal reference. Benefitting from the high photostability, well-separated spectrally-sharp emission, deep penetration, and high spatial resolution, this pair of ratiometric fluorescence probes enabled dual-channel NIR-II imaging of SLNs in 4T1 tumor-bearing mice in a non-invasive and sensitive manner 4. Notably, the obtained fluorescence ratiometric signal (I_1060 nm_/I_1525 nm_) could be used for real-time determination of the tumor-positive SLNs since this ratio significantly varies in non-metastatic and metastatic SLNs (**Scheme 1B-C**). Our developed NIR-II ratiometric fluorescence probes enable rapid and precise identification of the metastasis in SLNs, concurrently guiding the selective surgical removal of all tumor-infiltrating SLNs, which can avoid long-term complications of unnecessary dissection of normal SLNs, and therefore substantially improve the cancer surgery outcomes (**Scheme 1D**).

## Results and Discussion

### Design and synthesis of NIR-II ratiometric fluorescence probes

**Figure 1:**
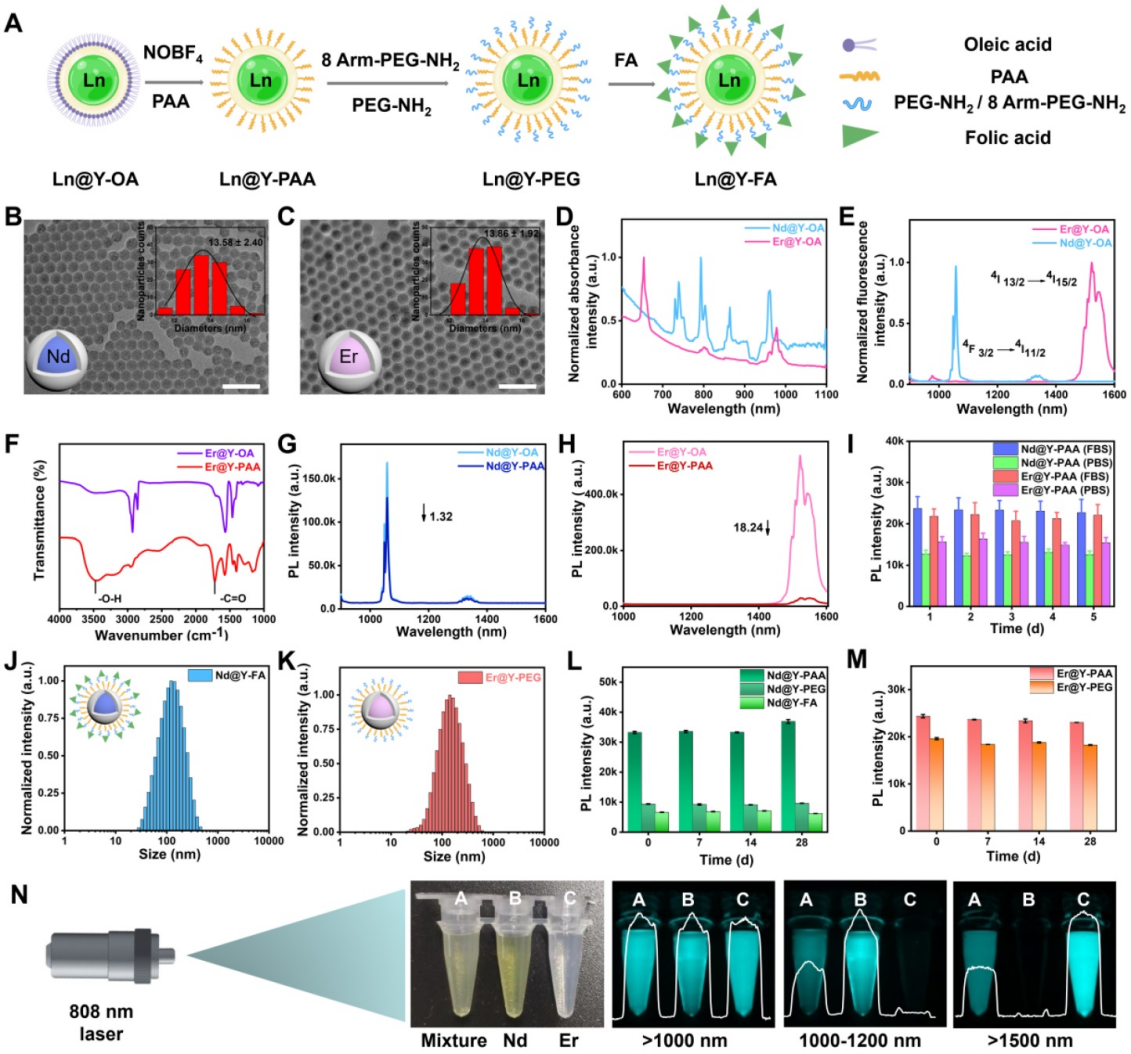
** Synthesis and characterization of the NIR-II ratiometric fluorescence probes.** (**A**) Schematic diagrams showing the surface modification of the NIR-II ratiometric fluorescence probes. Images were created with BioRender.com. (**B-C**) TEM images and Gaussian-fitted size distribution of as-made two types of core/shell nanocrystals. (**D**) The normalized absorption spectra of two NPs in hexane. (**E**) Fluorescence spectra of two NPs under the 808 nm excitation. (**F**) Fourier transform infrared spectroscopy of Nd@Y-PAA NPs and Er@Y-PAA NPs. (**G**) Luminescence spectra of oleic acid-capped Nd@Y NPs dispersed in hexane and the corresponding polyacrylic acid-modified Nd@Y NPs dispersed in PBS buffer. (**H**) Down-conversion emission spectra of the Er@Y-OA NPs in the organic environment and Er@Y-PAA NPs in the aqueous phase. (**I**) Emission intensity of Nd@Y-PAA NPs and Er@Y-PAA NPs in 1x PBS and FBS solution as a function of stored days. (**J-K**) Dynamic light scattering (DLS) results of Nd@Y-FA NPs and Er@Y-PEG NPs in MES buffer (pH = 8.5). (**L-M**) Long-term photostability of as-prepared NPs with different surface coating in PBS buffer. (**N**) Dual-NIR-II color imaging of Nd@Y-FA NPs, Er@Y-PEG NPs, and ratiometric fluorescence probes under >1000, 1000-1200, and >1500 nm windows, respectively.

To manufacture NIR-II ratiometric fluorescence probes, we initially synthesized two spectral non-overlapping lanthanide-doped downconversion nanoparticles (**Figure S1**) 51, and further devised Nd^3+^-doped nanoparticles with folic acid (FA) (Nd@Y-FA) as a targeting-moiety to provide a tumor-targeted imaging agent. Meanwhile, we also designed Er^3+^-doped nanoparticles by coating a cross-linking PEG polymeric layer (Er@Y-PEG) as a non-targeted ratiometric probe (**Figure 1A**). Suitable sizes of probes are essential for *in vivo* applications. Representative transmission electron microscopy (TEM) images of the Nd^3+^- or Er^3+^-doped core nanoparticles (NaYF_4_:Nd and NaErF_4_), and core-shell nanoparticles (NaYF_4_:Nd@NaYF_4_ and NaErF_4_@NaYF_4_) were presented in **Figure S2A,C** and **Figure 1B-C**, all showing the spherical shape and uniform size distribution. The mean sizes of Nd^3+^- and Er^3+^-doped core nanoparticles were measured to be 8.53 ± 0.96 and 9.69 ± 1.12 nm (**Figure S2**). Compared to the core nanoparticles, the sizes of their core/shell counterparts were 13.58 ± 2.40 nm and 13.86 ± 1.92 nm, suggesting that the shell layer has a thickness of ~2 nm. The recorded XRD patterns (**Figure S3**) confirmed the formation of pure *β*-phase Er-NPs and Nd-NPs. We then studied the optical properties of two lanthanide-doped nanoparticles, as it is necessary to determine their NIR-II performance. The absorption spectra showed representative inherent curves of Nd^3+^- and Er^3+^-doped core/shell nanoparticles (**Figure 1D**), while the emission spectra presented two non-overlapping downconversion NIR-II transitions. The NIR-II emission spans from 1060 nm (from Nd^3+^, 4F_3/2_ → 4I_11/2_), 1360 nm (from Nd^3+^, 4F_3/2_ → 4I_13/2_) to 1525 nm (from Er^3+^, 4I_13/2_ → 4I_15/2_) (**Figure 1E and Figure S4**) 52. The NIR-II fluorescence lifetimes of 1060 nm emission in Nd^3+^-doped nanoparticles and 1525 nm emission in Er^3+^-doped nanoparticles were 126.32 and 1686.03 μs, respectively, and these lifetimes kept constant in the mixture of Nd^3+^- and Er^3+^-doped nanoparticles solution, excluding the energy transfer and cross-talk between two nanoparticles (**Figure S5**). Collectively, the combined non-overlapping and consistent emission result in an efficient dual-fluorescence of lanthanide-doped nanoparticles towards NIR-II ratiometric fluorescence probes.

The as-prepared lanthanide-doped nanoparticles were capped with hydrophobic oleic acid ligands, which impeded their dispersion in an aqueous environment for biological delivery. In preparation for *in vivo* administration of ratiometric probes, we adopted a ligand-exchange protocol to replace the oleic acid on the surface of nanoparticles with an amphiphilic polyacrylic acid (PAA), imparting their water solubility (**Figure S6**) 53, 54. The successful modification of PAA was confirmed by the Fourier transform infrared spectroscopy (FTIR). An apparent new peak at 1720 cm^-1^ was ascribed to the typical vibration of the C=O stretching absorption band in the carboxyl group of PAA (**Figure 1F**). The average hydrodynamic diameters of PAA-coated Nd@Y NPs and PAA-coated Er@Y NPs were measured to be 58.8 and 68.06 nm, respectively (**Figure S7A-B**). When the NPs were transferred to the aqueous phase, it was worth noting that the NIR-II fluorescence intensity at 1060 nm from Nd@Y NPs showed a ~1.32-fold decrease while Er@Y NPs with 1525 nm fluorescence emission displayed an 18.24-fold brightness depletion (**Figure 1G-H**). The NIR-II fluorescence intensity attenuation is presumably caused by the energy transfer from the emitting energy levels of NPs to the vibronic energy of the hydroxyl group of water molecules. Nevertheless, the NIR-II brightness of NPs is still sufficient for *in vivo* visualization even with the disparate depletion. Examining the photostability of two NPs under continuous 808 nm laser irradiation for 2 h results in no apparent signal decline for several aliquots of PAA-coated NPs with different concentrations (**Figure S8**). Moreover, the long-term stabilities of PAA-coated Nd@Y NPs (Nd@Y-PAA) and Er@Y NPs (Er@Y-PAA) were verified by monitoring the NIR-II brightness after storing them in PBS and FBS for over 5 days, and the plotted result indicates that these NPs are very stable for long-term storage and *in vivo* illumination (**Figure 1I**).

With the water-soluble lanthanide-doped nanoparticles in hand, we further grafted a polymeric layer of mixed methoxy polyethylene glycol amine (mPEG-NH_2_) and 8 Arm-PEG-NH_2_ (ratio = 1:5, see methods in Supplementary Material) on both two PAA-coated nanoparticles, aiming to form high cross-linking hydrophilic polymeric layers and simultaneously impart amine groups for the following targeting-moiety conjugation (**Figure S6**) 55-57. To achieve the targeted components of NIR-II ratiometric probes, we conjugated FA (a small molecule for targeting folate receptors) to PEGylated Nd^3+^-doped nanoparticles through 1-(3-dimethylaminopropyl)-3-ethylcarbodiimide hydrochloride (EDC) chemistry (see methods in Supplementary Material). Successful conjugation of FA was confirmed by Zeta potential measurement (**Figure S7C**) and Fourier transform infrared spectroscopy (FTIR) (**Figure S9**). Meanwhile, the PEGylated Er^3+^-doped nanoparticles were selected as untargeted components to form the ratiometric pair. Dynamic light scattering (DLS) results illustrated that the overall hydrodynamic sizes of Nd@Y-FA NPs and Er@Y-PEG NPs were ~142 and 141.8 nm, respectively (**Figure 1J-K**). Although the NIR-II brightness decreased from the original oleic acid-coated NPs to the final three layers-coated NPs, we are most impressed by their remarkable fluorescence stability even over 4-weeks of monitoring, primed for *in vivo* ratiometric fluorescence imaging (**Figure 1L-M**).

We further verified the NIR-II ratiometric fluorescence imaging ability by mixing the final optimized Nd@Y-FA NPs and Er@Y-PEG NPs with equivalent fluorescence intensity under their corresponding emission filters. To quantify the dosage information of nanoparticles, we first investigated the correlation between the concentration and fluorescence intensity in Nd@Y-FA NPs and Er@Y-PEG NPs. Plotting the fluorescence intensity of two nanoparticles in series against concentration resulted in a good linear relationship, which thus provides an accurate database for quantifying dosage information (**Figure S10**). Imaging of tubes containing Nd@Y-FA NPs (A, left), Er@Y-PEG NPs (B, center), and mixed nanoparticles (C, right) in real time was conducted under >1000, 1000-1200, and >1500 nm sub-NIR-II windows. The NIR-II fluorescence signals at 1060 nm and 1525 nm with diverse channels were clearly distinguished, confirming the non-overlapping emission of agents (**Figure 1N**). In addition, we evaluated the biosafety of these nanoparticles, which showed negligible toxicities to 4T1 breast cancer cell and L929 fibroblasts cell lines even at a very high dose of 292 mg/mL (**Figure S11**).

### Visualization of normal and metastatic SLNs based on NIR-II ratiometric fluorescence

**Figure 2:**
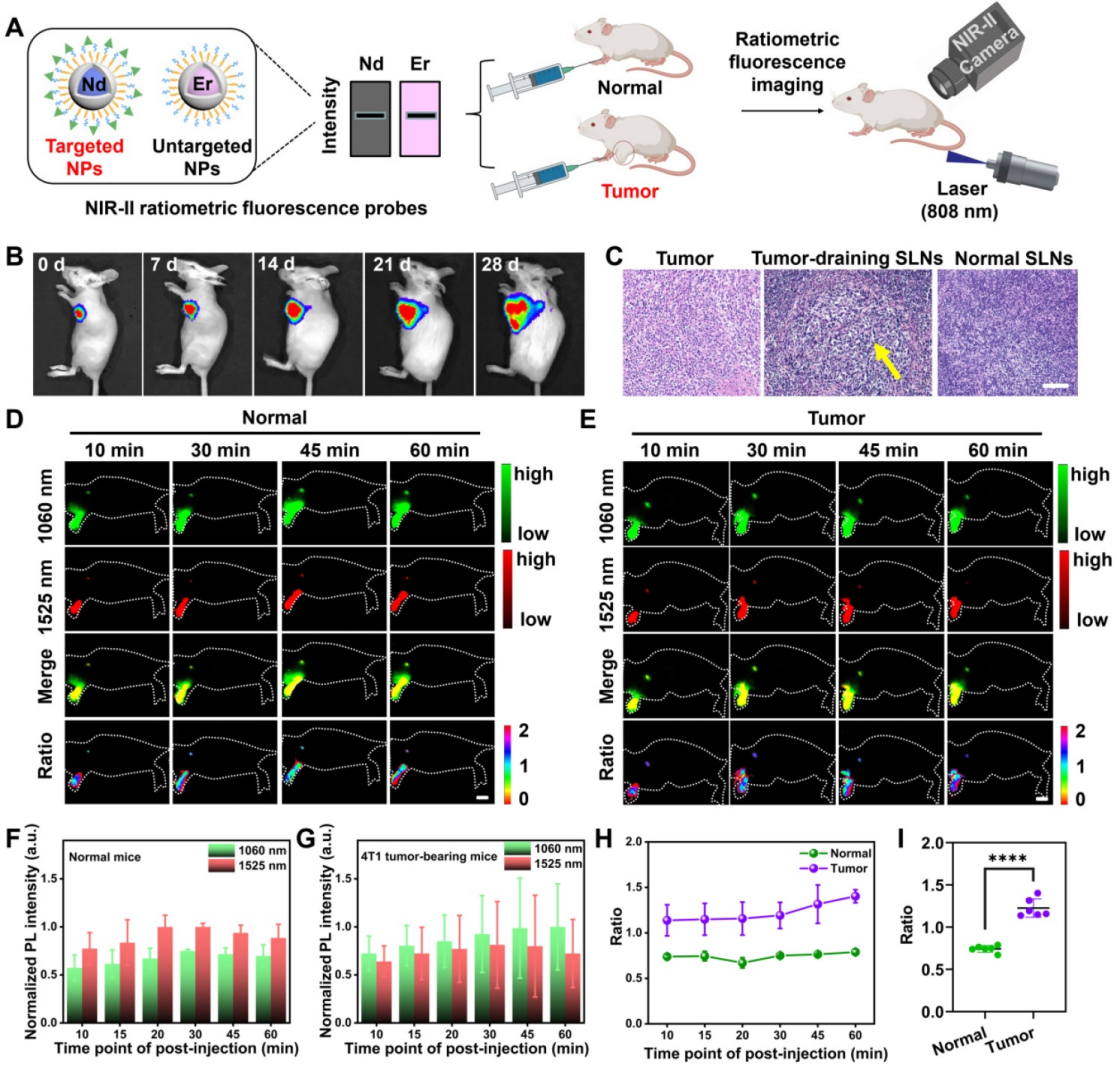
***In vivo* dual-channel NIR-II fluorescence imaging for mapping SLNs.** (**A**) Schematic illustration outlining NIR-II ratiometric fluorescence imaging of SLNs in two animal models using probes. Images were created with BioRender.com. (**B**) Tumor progression and metastasis to SLNs were monitored by the bioluminescence of luciferase-transfected 4T1 cells. (**C**) H&E staining of excised SLNs and tumors verified the presence of metastasis in SLNs. (**D-E**) *In vivo* NIR-II fluorescence images under two channels and the corresponding merging and ratio images of SLNs in normal and tumor-metastatic mice excited by a single 808 nm laser (65 mW/cm^2^, 100 ms). Scale bar: 1 cm. The normalized fluorescence intensities from 1060 and 1525 nm channels of SLNs in normal mice (**F**) and tumor metastatic mice (**G**) were plotted against different post-injection time points. The error bars represented mean ± SD are generated from n = 3 biologically independent mice for each group. The collected signals were further normalized by the highest fluorescence intensity among the post-injection time-points. (**H**) NIR-II ratiometric signals of I_1060 nm_/I_1525 nm_ of SLNs were obtained from two cohorts of normal and tumor metastatic mice over time. (**I**) Evaluation of the ratiometric signals of two groups. A lower value was observed for normal groups. The bars represent ± SD, ****P < 0.0001.

For non-invasively mapping SLNs, we performed NIR-II ratiometric fluorescence imaging with as-prepared probes in both normal healthy mice and SLN-metastatic 4T1 tumor-bearing mice (**Figure 2A**). To this end, we first established the tumor model by inoculating luciferase-transfected 4T1 cells into the mammary fat pad of mice 58, and the tumor growth was monitored by bioluminescence imaging (BLI). After 4 weeks, the signal distribution of bioluminescence indicated that the 4T1 cells in the primary tumor had migrated to SLNs, which was further confirmed by pathological examination (**Figure 2B-C**). Then, we intradermally injected the NIR-II fluorescence probes into the footpads of mice and monitored the SLNs with a customized NIR-II imaging set-up. As shown in **Figure 2D-E**, SLNs of the healthy mice and tumor-bearing mice were visualized by the as-prepared probes in two sub-NIR-II windows. The probes illuminated the SLNs in 10 min and reached their maximum accumulations in 20-30 min. The stable NIR-II signal was exclusively located in tumor-adjacent LNs (sentinel or higher-tier LNs), with extremely low off-target signals over any position of the body. Taken together, the stable fluorescence signal output was necessary to enable effective SLNs mapping.

Notably, due to the reduced tissue scattering and autofluorescence, even with identical injected dosage (total brightness) of two nanoprobes (**Figure 2A**), the NIR-II signal collected from the 1500 nm emissive Er@Y-PEG was always higher than that of the 1060 nm Nd@Y-FA emissive probe. Therefore, plotting the SLNs signals from two windows against post-injection time points indicated that NIR-II fluorescence intensity at 1060 nm from Nd@Y-FA was always lower than that of Er@Y-PEG at 1525 nm in a cohort of health mice (**Figure 2D, F**). Conversely, for the cohort of mice with tumor draining into SLNs, the SLNs signal of the 1060 nm channel was higher than that of the 1525 nm channel, which is ascribed to the increased accumulation of targeting Nd@Y-FA NPs in tumor-draining SLNs compared with normal nodes (**Figure 2E-G**). Taking the emission at 1525 nm as a (self-calibrated) reference signal, we further utilized the ratio of the collected two NIR-II fluorescence signals (I_1060 nm_/I_1525 nm_) to intuitively identify metastatic and normal SLNs, and the calculated ratiometric signal of metastatic SLNs was significantly higher than that of normal SLNs (**Figure 2H-I**). These data therefore inspired us to use NIR-II ratiometric fluorescence probes to differentiate normal SLNs from abnormal ones through a rapid, real-time, and non-invasive approach. Although single administration of Nd@Y-FA NPs could also target the tumor-draining SLNs, the single fluorescence signal failed to provide a reliable, accurate diagnosis and the detection needs to compare with normal SLNs to provide staging information (**Figure S12**). It is worth mentioning that the surface binding of folic acid has moderate influence on the mobility rate of these intradermally injected probes from injected sites to adjacent SLNs (**Figure S13**). To further demonstrate the proof-of-concept and availability of our ratiometric fluorescence platform, we manufactured an additional ratiometric probe pair with Er@Y-FA NPs and Nd@Y-PEG NPs, in which the targeting-moiety FA was switched from Nd to Er NPs. In the *in vivo* testing, the ratiometric fluorescence of I_1525 nm_/I_1060 nm_ also allowed us to discriminate between metastatic and non-metastatic SLNs (**Figure S14**). After establishing the ratiometric tumor-targeting contrast agent to identify metastasis, the next goal was to provide a rapid/accurate/non-invasive intraoperative decision-making of sentinel and/or higher-tier LNs for image-guided surgery that can substantially benefit the clinical treatment strategy of solid tumors.

### Identification mechanism and blind-test of metastatic SLNs based on NIR-II ratiometric fluorescence

**Figure 3:**
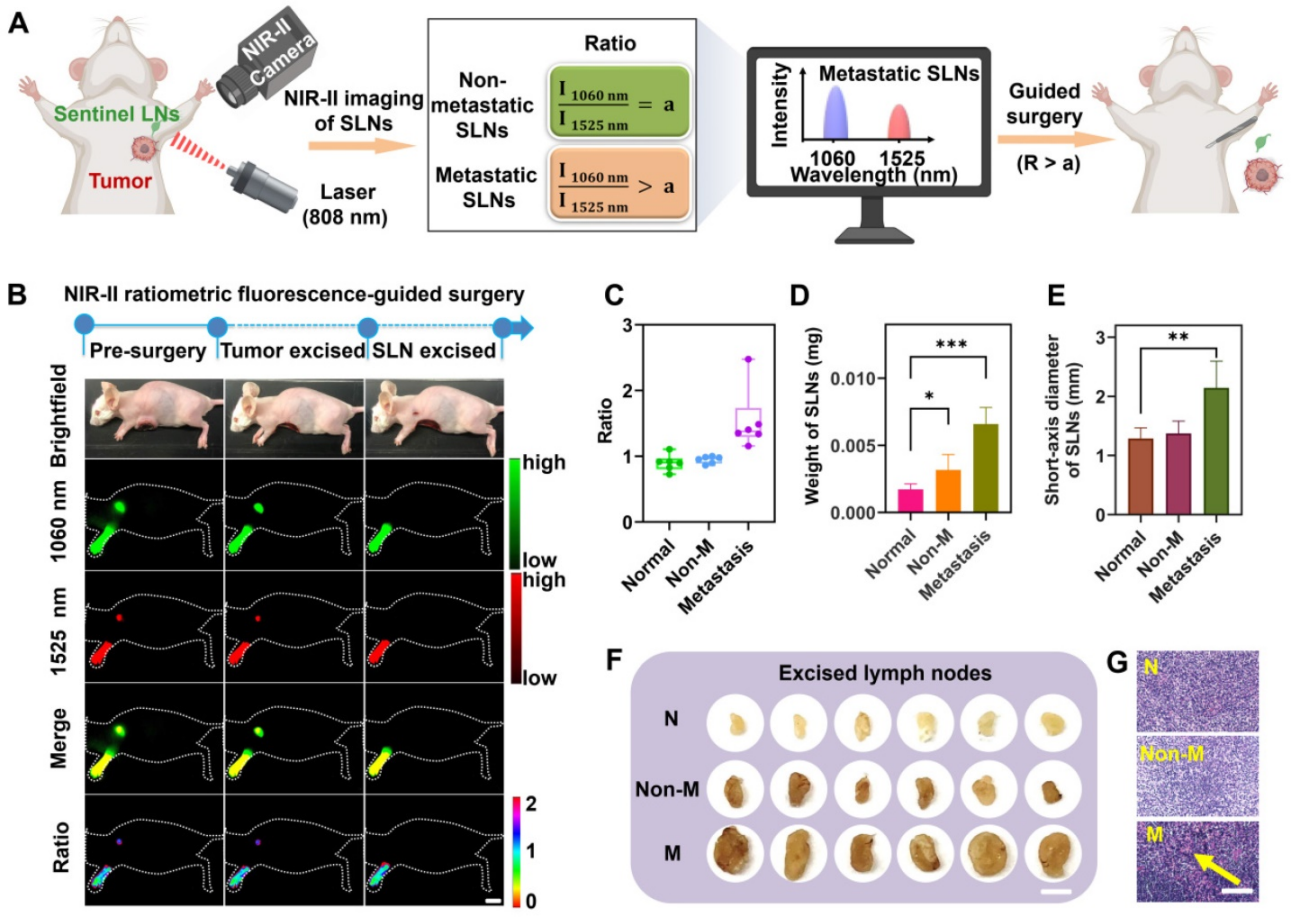
** Identification of the metastatic state of SLNs based on NIR-II ratiometric fluorescence.** (**A**) Scheme of NIR-II ratiometric fluorescence probes for intraoperative detection and guided-surgery in orthotopic 4T1 breast cancer model. Images were created with BioRender.com. (**B**) NIR-II ratiometric fluorescence strategy for preoperative diagnosis and intraoperative navigation surgery in an orthotopic 4T1 breast cancer model. (**C**) Ratiometric signals (I_1060 nm_/I_1525 nm_) of SLNs in different groups, including (i) normal SLNs, (ii) non-metastatic SLNs, and (iii) metastatic SLNs (n = 6 for each group). (**D**) The weight of SLNs in all three groups (n = 6 for each group). Error bars mean ± SD, *P < 0.05, ***P < 0.001. (**E**) Short-axis diameter of SLNs in different groups (n = 6 for each group). Error bars mean ± SD, **P < 0.01. (**F**) Photograph of excised SLNs from three groups. N represents normal SLNs, Non-M represents non-metastatic SLNs, and M represents metastatic SLNs. The scale bar is 20 mm. (**G**) Representative H&E staining images of SLNs derived from different groups. Yellow arrows indicate foci occupied by tumor cells. The scale bar is 100 µm.

We then sought to understand the mechanism of the NIR-II ratiometric fluorescence probes for metastatic SLN identification (**Figure 3A** and **Figure S15A**). In the SLNs imaging, two non-overlapping signals from the ratiometric probes were consecutively captured by the NIR-II camera under two combinations of emission filters. For normal SLNs, the targeted Nd@Y-FA NPs and non-targeted Er@Y-PEG NPs had similar migration and distribution, conversely, for mice with tumor-infiltrating SLNs, the accumulation of Nd@Y-FA NPs would increase significantly due to the specific binding of FA to folate receptor expressed on tumor cells. Thus, the ratio of fluorescence intensities from 1060 nm and 1525 nm (I_1060 nm_/I_1525 nm_), also represented as NIR-II ratiometric fluorescence, would offer a new strategy to identify the metastatic status of SLNs. Depending on the clinical need, only the tumor-positive SLNs need to be excised by NIR-II fluorescence navigation, thereby saving those normal nodes that are regularly lost in radical lymphadenectomy.

To mimic the clinical situation in breast cancer surgery, three cohorts of normal mice, non-metastatic (tumor-negative SLNs) tumor-bearing mice, and metastatic (tumor-positive SLNs) tumor-bearing mice were randomly caged and separated for blind-test and navigation surgery. After administration of our nanoprobe cocktails, the NIR-II ratiometric examination was collected within the screened optimal time point (30 min p.i. according to result from the **Figure 2F**). The NIR-II fluorescence-guided surgery was only performed on those mice with positive ratio values (**Figure 3B**), while the normal LNs were saved for mice with negative ratio values (**Figure S15**). The blindly tested results were plotted in **Figure 3C**, which were consistent with the pathological examination of H&E staining results (**Figure 3G**). The collected ratiometric signals (I_1060 nm_/I_1525 nm_) showed approximately the same range in normal healthy SLNs (0.90 ± 0.12) and non-metastatic SLNs (0.95 ± 0.05). In sharp contrast, this value in metastatic SLNs was observably higher than in the other groups, demonstrating the NIR-II ratiometric fluorescence could effectively identify the metastatic SLNs. After excision of SLNs, we compared the morphology, weight, and size of harvested SLNs in different groups, and found that metastatic SLNs were obviously enlarged, which could be attributed to the 4T1 tumor cell migration and progression (**Figure 3D-F**). Collectively, these results suggested that the effectiveness of the NIR-II ratiometric fluorescence probes for SLNs identification could be rationally illustrated by the distinct uptake ability of two types of probes in different SLNs.

### The advantage of predicting the metastatic status of SLNs

**Figure 4:**
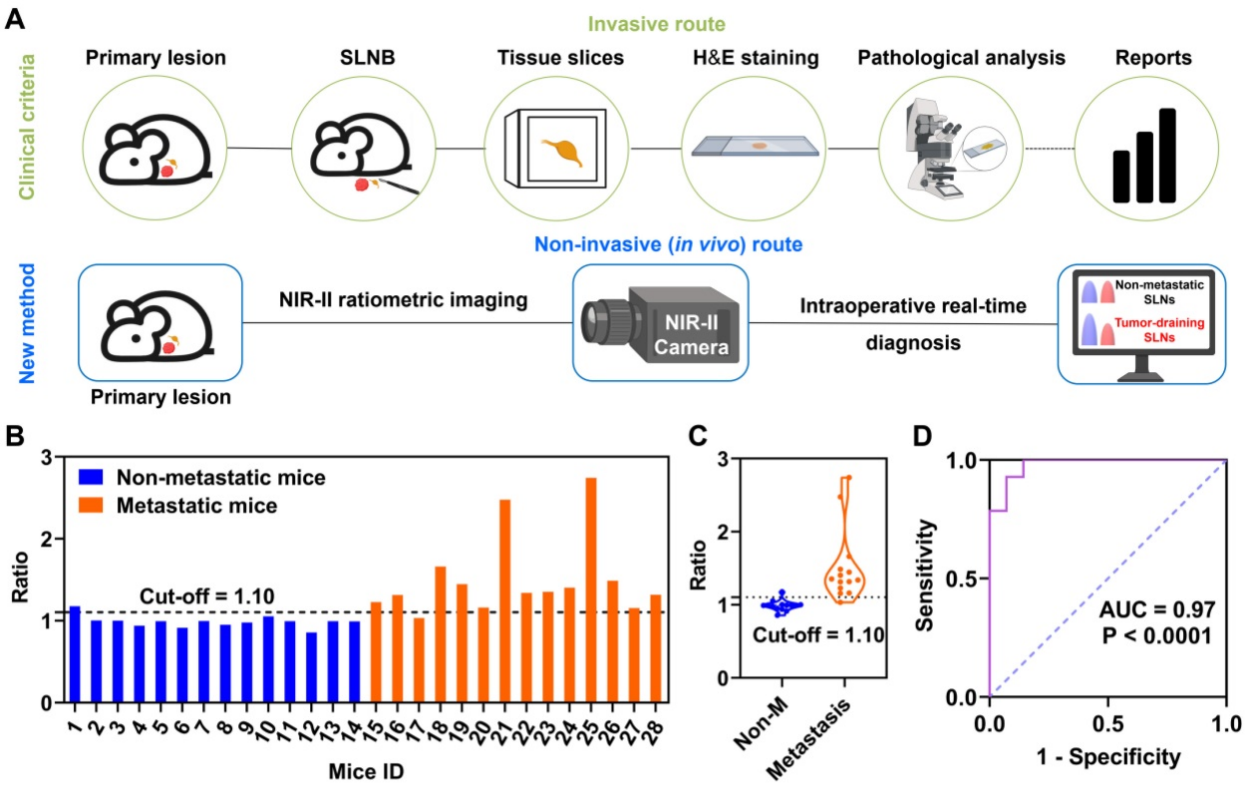
** The advantage of NIR-II ratiometric fluorescence strategy for intraoperatively diagnosing metastatic SLNs.** (**A**) Two methods for identifying metastatic SLNs in orthotopic 4T1 breast cancer. The clinical pathological analysis is an invasive method while our new method is a real-time and non-invasive strategy. Images were created with BioRender.com. (**B**) Identification of metastatic SLNs based on ratiometric fluorescence signals. Blue bars represent 4T1 tumor-bearing mice with no tumor cell metastasizing in SLNs while the orange histograms represent 4T1 tumor-bearing mice with tumor-metastatic SLNs (n = 14 for each group). (**C**) Ratios of I_1060 nm_/I_1525 nm_ in both groups (Non-M represents non-metastatic SLNs; M represents metastatic SLNs; n = 14 for each group). Error bars represent ± SD. (**D**) ROC curve for recognition of metastatic SLNs based on NIR-II ratiometric fluorescence strategy. When the cut-off value of the ratio is set to 1.10, the AUC is 0.97.

Pathological assessment is widely used in clinic to assess tumor expression and provide intraoperative decision-making. For example, the diagnostic gold standard H&E staining needs an invasive LN excision route, which also suffers from complex and time-consuming operations as well as needs expert assessment by a well-trained pathologist. Conversely, our developments of the NIR-II strategy offer a rapid, non-invasive, and real-time diagnosis (**Figure 4A**). In addition, the advantage of our NIR-II ratiometric fluorescence imaging is that all procedures can be performed under the ambient white light in the operating room without changing the current surgical settings. Thus, our established NIR-II fluorescence imaging with ratiometric nanoprobes outperformed the clinical H&E staining with a better, faster, and more accurate procedure.

We further evaluated the detection accuracy of our ratiometric approach in a cohort of mice containing tumor-positive SLNs (n = 14) and tumor-negative SLNs (n = 14). Receiver operating characteristic (ROC) curve analysis was collected according to the NIR-II ratiometric fluorescence signals (I_1060 nm_/I_1525 nm_) for the 14 negative and 14 tumor-positive metastatic SLNs (**Figure 4D**). The cut-off values were determined to be 1.10 with a specificity of 0.97, which could offer sufficient sensitivity for detecting the metastatic status (**Figure 4B-C**). Moreover, only one mouse with negative SLN was found to be false-positive and the final accuracy rate was 92.8% (26/28). These results indicated that our NIR-II imaging agents enabled an effective detection of metastatic SLNs with ratiometric fluorescence signals, which presented a relatively high accuracy rate and non-invasive signal output compared with clinical pathological assessment. Our strategy will substantially improve the current fluorescence-guided surgery outcomes, which therefore warrants more preclinical and clinical studies.

### NIR-II ratiometric fluorescence imaging enabled precise navigation surgery of breast cancer

**Figure 5:**
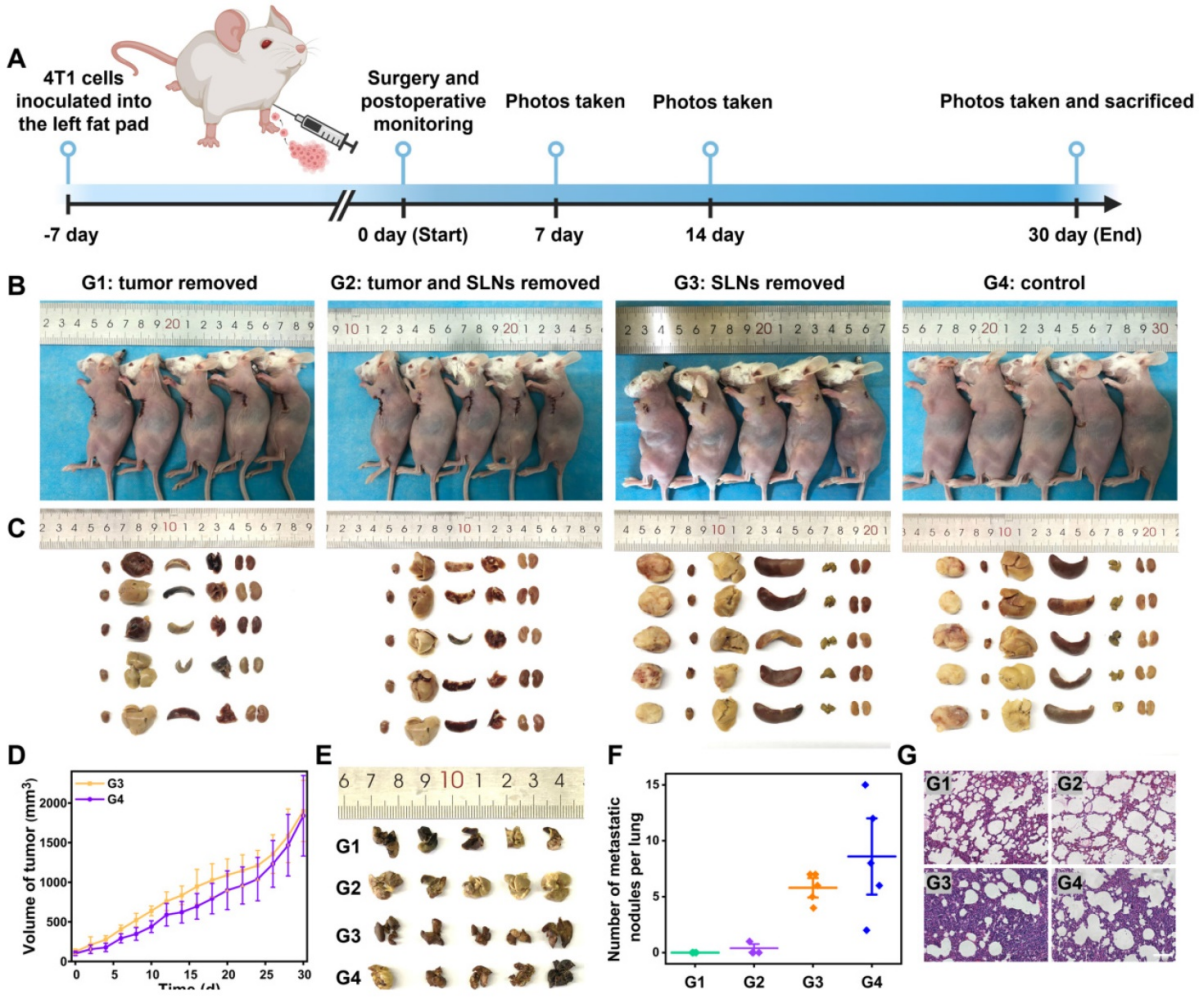
The improved surgery outcomes of 4T1 tumor-bearing mice with NIR-II ratiometric imaging-guided identification and resection of the SLNs. **(A)** Schematic workflow for detecting and treating orthotopic 4T1 breast tumor by using the NIR-II ratiometric navigation system. Images were created with BioRender.com. **(B)** Representative photographs of 4T1-bearing Balb/c mice treated with different surgeries. Group 1: the tumor was removed; Group 2: tumor and SLNs were removed; Group 3: SLNs were removed; Group 4: no treatment as the control. **(C)** Pictures of excised tumor and main organs (heart, liver, spleen, lung, and kidneys) derived from the above four cohorts of mice. **(D)** Tumor growth curves of 4T1-bearing mice in groups 3 and 4 (n = 5/group). **(E)** Representative 3D volume visualization of excised lungs (n = 5/group). **(F)** Analysis of lung metastasis nodes. **(G)** H&E staining images of lungs from all groups. The scale bar is 100 µm.

The detection of tumor-positive SLNs is essential in cancer surgery that determines tumor staging, treatment, and prognosis. Current fluorescence-guided surgeries with tumor-targeted imaging contrast agents are not always effective for discriminating between metastatic and normal nodes 37, 59. To investigate whether surgical treatment guided by our intraoperative SLNs identification approach could increase the survival outcomes in tumor-bearing mice, we performed the NIR-II ratiometric fluorescence imaging to confirm the “present” and “absent” tumor-infiltrating SLNs, followed by image-guided surgery for only primary tumor and the tumor-positive SLNs, after that, the postoperative recovery was monitored over time (**Figure 5A**). The cohort of mice with the unknown metastatic status of SLNs was established and *in vivo* detected by NIR-II ratiometric fluorescence signals. Based on the outcomes of SLNs identification, all tumor-bearing mice were diagnosed with non-metastatic SLNs (consistent with the tumor inoculation period). As shown in **Figure 5B**, we further randomly divided mice into four groups for three types of treatments, including: Group 1) only tumor excision, Group 2) tumor and SLNs excision, and Group 3) only SLNs excision; while Group 4) tumor-bearing mice without any treatment were set as a negative control group. After finishing the corresponding surgery for each group, we collected mouse photos once a week and measured the tumor volume every two days (**Figure 5B, D** and **Figure S16**). Notably, the primary tumors disappeared with no recurrence in Groups 1 and 2, while mice in Groups 3 and 4 suffered from tumor progression and recurrence, and even one death occurred at day 28 in Group 3. According to the similar treatment outcomes in Groups 1 and 2, it can be concluded that the resection of tumor-negative SLNs is not necessary. Our non-invasive diagnosis therefore will substantially lower the rate of complications that are always caused by the current clinical workflow.

We further compared the surgery outcomes through testing pathological indicators. Mice from four groups were sacrificed on the 30th day after the image-guided surgeries, and SLNs as well as all main organs (heart, liver, spleen, lung, and kidneys) were collected for pathological analysis (**Figure 5C** and **Figure S17**). The tumor volumes in Groups 3 and 4 increased over time (**Figure 5D**), which was consistent with images and weights of the harvested tumors *ex vivo*. Previous reports have proven that 4T1 breast cancer cells are highly invasive and could migrate to the lung 60. We thus stained the harvested lungs from the four groups to examine the tumor metastasis (**Figure 5E-F**), and the results indicated that Groups 3 and 4 exhibited obvious lung metastatic loci. Additionally, the H&E staining also confirmed the unsuccessful treatment outcomes and the lung metastasis in Groups 3 and 4 (**Figure 5G**). Taken together, our strategy to identify the tumor-positive SLNs during the surgery provides intraoperative planning of resection, which could guide rational treatment procedures with greatly improved surgical outcomes.

## Conclusion

In conclusion, we combined two NIR-II ratiometric fluorescence probes as a novel tumor-targeted contrast agent. The NIR-II fluorescence imaging in two non-overlapping channels showing ratiometric fluorescence signals can effectively map SLNs and discriminate tumor-draining SLNs from normal nodes. The collected NIR-II ratiometric fluorescence signals (I_1060 nm_/I_1525 nm_) could also be used to identify the metastatic SLNs because of the significantly higher accumulation of targeting probes in tumor-positive SLNs. This non-invasive detection approach not only provides high diagnostic accuracy, which is significantly better than pathological diagnosis in clinic, but also enables a time-saving operation procedure, which outperforms the current gold standard paraffin section for SLN identification. Our strategy will not alter the settings of the current surgical field since the NIR-II imaging can be performed under the operating light 61. Therefore, the NIR-II ratiometric fluorescence probes can facilitate an ideal image-guided surgery for a variety of cancers, particularly for breast cancer 62. The precise identification of tumor-infiltrating SLNs in a real-time manner will have the potential to replace the current clinical standard of invasive pathological assessment, avoiding the unnecessary removal of non-metastatic SLNs and substantially improving surgical resection outcomes.

## Methods

### Preparation of NIR-II Ratiometric Fluorescence Probes

The core-shell nanoparticles of NaYF_4_:Nd@NaYF_4_ and NaErF_4_@NaYF_4_ were prepared and modified following the previously reported method 51, 53, 56. The synthesis protocols and details were provided in the Supplementary Material. NIR-II ratiometric fluorescence probes were prepared by mixing the final optimized Nd@Y-FA NPs and Er@Y-PEG NPs with equivalent fluorescence intensities under the InGaAs camera (Princeton Instruments, NIRvana-640).

### Animals and Tumor Model

All animal experiments were conducted by the institutional guidelines and were approved by the Experimental Animal Ethical Committee of the First Hospital of Jilin University (Protocol number: 20210642). Six-week-old Balb/c female mice were purchased from Liaoning Changsheng biotechnology Co. Lt.

Orthotopic tumors were established by inoculating ~0.5 × 10^6^ tumor cells into the second mammary fat pad. The tumor growth was monitored by bioluminescence imaging with an IVIS Lumina III (PerkinElmer) system after intraperitoneal injection of 100 μL (20 mg/mL) luciferase. After ∼4 weeks, the bioluminescence spread around the tumor was detectable and mice were used for further experiments.

### NIR-II Ratiometric Fluorescence Imaging of SLNs

All mice were shaved using Nair hair removal lotion and anesthetized with isoflurane before the experiment. ~25 μL NIR-II fluorescence probes (146 mg/mL) were injected into the footpads of mice to visualize SLNs. Then the NIR-II SLNs imaging were performed under an imaging condition of an 808 nm laser excitation, ~65 mW/cm^2^ power density, and 100 ms exposure time. All NIR-II images were collected on a two-dimensional InGaAs camera. The fluorescence signals of Er@Y-PEG NPs were collected in >1500 nm region and signals of Nd@Y-FA NPs were collected in 1000-1200 nm region.

### Metastatic SLNs Detection

According to the NIR-II fluorescence imaging of SLNs, 1060 nm and 1525 nm fluorescence signals were separately collected at 30 min post-injection under a single 808 nm excitation. The NIR-II ratiometric fluorescence signals (I_1060 nm_/I_1525 nm_) were quantified for real-time determination of the tumor-positive SLNs. The unknown metastatic SLNs in tumor-bearing mice were identified by NIR-II ratiometric fluorescence imaging for a blind-test. The mice with negative ratio values only received tumor resection, while the metastatic SLNs were further resected for the mice with positive ratio values.

### Precise Navigation Surgery of Metastatic SLNs

Sterile surgical instruments were prepared prior to surgery. The tumor mice with positive ratio values were anesthetized under isoflurane and placed on the plate. Then, the resection of metastatic SLNs was performed under the ratiometric fluorescence guidance. To further verify surgery outcomes by precise navigation, four groups of mice, including only tumor excision, tumor and SLNs excision, only SLNs excision, and tumor-bearing mice without any treatment, were monitored with appearance (digital photos), weight, and tumor volume for one month.

## Supplementary Material

Supplementary methods and figures.

## Abbreviations

NIR-II: near-infrared-II
SLNs: sentinel lymph nodes
FGS: fluorescence-guided surgery
TEM: transmission electron microscopy
EDC: 1-(3-dimethylaminopropyl)-3-ethylcarbodiimide hydrochloride
DLS: dynamic light scattering
BLI: bioluminescence imaging
ROC: receiver operating characteristic
