# Supplementary material for: Near-infrared-II ratiometric fluorescence probes for non-invasive detection and precise navigation surgery of metastatic sentinel lymph nodes
Source: Theranostics. 2022 Oct 9;12(16):7191–202. doi: 10.7150/thno.78085 (PMC9576618; doi:10.7150/thno.78085)
Supplement: Supplementary file 1 — Supplementary methods and figures. [file thnov12p7191s1.pdf]

Supplementary Material

Near-infrared-II ratiometric fluorescence probes for non-invasive detection and precise navigation surgery of metastatic sentinel lymph nodes

Mengfei Li [1,2], *Xue Zheng* [1,2], *Tianyang Han* [2], *Shengjie Ma* [3], *Yajun Wang* [2], *Bin Sun* [1,2], *Jiajun Xu* [2], *Xin Wang* [1,4]*, *Songling Zhang* [4]*, *Shoujun Zhu* [1,2]*, *Xiaoyuan Chen* [5,6,7]*

[1] Joint Laboratory of Opto-Functional Theranostics in Medicine and Chemistry, The First Hospital of Jilin University, Changchun, 130021, China

[2] State Key Laboratory of Supramolecular Structure and Materials, College of Chemistry, Jilin University, Changchun 130012, China

[3] Department of Gastrointestinal Surgery, The First Hospital of Jilin University, Changchun, China

[4] Department of Obstetrics and Gynecology, The First Hospital of Jilin University, Changchun, 130021, China

[5] Departments of Diagnostic Radiology, Surgery, Chemical and Biomolecular Engineering, and Biomedical Engineering, Yong Loo Lin School of Medicine and Faculty of Engineering, National University of Singapore, Singapore, 119074, Singapore

[6] Clinical Imaging Research Centre, Centre for Translational Medicine, Yong Loo Lin School of Medicine, National University of Singapore, Singapore 117599, Singapore

[7] Nanomedicine Translational Research Program, NUS Center for Nanomedicine, Yong Loo Lin School of Medicine, National University of Singapore, Singapore 117597, Singapore

Supplementary Methods……………………………………………………………Pages 3-7

Supplementary Figures……………………………………………………………Pages 8-22

Supplementary Methods

Materials

Erbium (III) chloride hexahydrate ($ErCl_3 \cdot 6H_2O$, 99.99%), Neodymium (III) chloride hexahydrate ($NdCl_3 \cdot 6H_2O$, 99.99%), Yttrium oxide (Y_2O_3, 99.99%), sodium trifluoroacetate (CF_3COONa, 98%), trifluoroacetic acid (TFA), sodium oleate (Na-OA, >97%), N-hydroxysulfosuccinimide sodium salt ($NOBF_4$, 97%), folic acid, dichloromethane, hexane, toluene, chloroform, acetone, N,N-Dimethylformamide (DMF), and polyacrylic acid (PAA, Mw = 3000) were all purchased from Aladdin. Oleic acid (OA, tech grade, 90%), 1-octadecene (ODE, tech grade, 90%), 1-(3-dimethylaminopropyl)-3-ethylcarbodiimide hydrochloride (EDC), 4-morpholineethanesulfonic acid (MES), and oleyl amine (OM, tech grade, 70%) were purchased from Sigma-Aldrich. The mPEG-NH_2 (average molecular weight: 5,000) and 8Arm-PEG-NH_2 (average molecular weight: 40,000) were purchased from Xi'an Ruixi Biological Technology Co., Ltd. All chemicals were used as received without further purification.

Characterizations

Transmission electron microscope was conducted using a JEM-2100F. The powder X-ray diffraction (XRD) patterns of as-prepared NPs were measured on a Bruker D8 ADVANCE at a scanning speed of 7°/min. Dynamic light scattering (DLS) and zeta potential measurements were tested using a Nano Zetasizer system (Nano ZS ZEN3600, Malvern). Fourier transform infrared spectroscopy (FTIR) technique was recorded with IFS-66V/S spectrometer (Bruker, Germany). PerkinElmer Lambda 950 was employed to measure the ultraviolet-visible-near infrared (UV-VIS-NIR) absorption spectra. Fluorescent emission spectra were collected by an Edinburgh FL 920 spectrofluorometer equipped with 808 nm diode lasers.

Synthesis of β-$NaErF_4$ NPs.

Tiny *β*-$NaErF_4$ NPs were prepared following the previously reported method with adaptations [1]. Firstly, the RE-OA precursor was prepared using the following procedure. $ErCl_3\cdot 6H_2O$ (1 mmol) and 3 mmol sodium oleate were mixed with 3 mL of deionized water, 3.5 mL of absolute ethyl alcohol, 7 mL of hexane, and the resulting mixture was heated at 60 °C overnight. The organic phase solvent containing Er-OA was collected through a separatory funnel and washed three times against with deionized water. Secondly, the obtained Er-OA precursor was mixed with 4 mmol sodium oleate, 5.2 mL of OA, 5.1 mL of OM, and 9 mL of ODE. The solution was then heated up to 100 °C under argon gas protection with vigorous magnetic stirring for 60 min. Subsequently, 4 mmol solid ammonium fluoride was added to the solution and kept at 100 °C for another 30 min. Lastly, the reaction mixture was heated to 300 °C at a rate of 10 $K\cdot min^{-1}$, kept at this temperature for 30 min, and then allowed to cool down to room temperature naturally. The resulting *β*-$NaErF_4$ was precipitated by the addition of 20 mL of ethanol and collected via centrifugation at 6000 rpm for 10 min, followed by washing twice against a 1:6 hexane/ethanol mixture, and finally the product was dispersed in 10 mL of hexane for further use.

Synthesis of *β*-$NaErF_4$@$NaYF_4$ NPs

Typically, 1 mmol Y_2O_3 was first dissolved in 10 mL of TFA solution of 50% (v/v) under heating at 90 °C in a three-neck flask. After the solution became transparent, the solution was evaporated to dryness under argon gas protection, yielding the precursor $(CF_3COO)_3Y$. Subsequently, 10 mL of OA, 10 mL of ODE, 2 mmol sodium trifluoroacetate, 1 mM $(CF_3COO)_3Y$, and 5 mL of hexane with dispersed *β*-Er NPs were loaded into a three-neck flask. The mixture was then heated to 120 °C for 30 min, and then to 300 °C at a rate of 10 $K\cdot min^{-1}$ under argon gas protection. After heating at 300 °C for 30 min, the reaction mixture was allowed to naturally cool down to room temperature. The resulting core/shell NPs were collected using the procedure which was

described above for the β-$NaErF_4$ NPs. These NPs were then dispersed in 10 mL of hexane for further use.**Synthesis of β-$NaYF_4$:Nd NPs**

The procedure to synthesize β-$NaYF_4$:Nd NPs is almost identical to that for β-$NaErF_4$ NPs. The difference is to use $YCl_3 \cdot 6H_2O$ (0.95 mmol) and $NdCl_3 \cdot 6H_2O$ (0.05 mmol) to substitute $NdCl_3 \cdot 6H_2O$ (1 mmol).

Synthesis of β-$NaYF_4$:Nd@$NaYF_4$ NPs

The procedure to synthesize β-$NaYF_4$:Nd@$NaYF_4$ core/shell NPs is similar to that for β-$NaErF_4$@$NaYF_4$ NPs. The difference is to use 5 mL of β-$NaYF_4$:Nd NPs to replace 5 mL of β-Er NPs as the seeding core.

Surface Modification of as-prepared NPs

The oleic acid ligand of as-prepared NP is hydrophobic, which is replaced using a hydrophilic ligand of PAA to render them water dispersible. The ligand exchange was adapted from a literature method [2, 3]. Typically, 2 mL of as-prepared NPs dispersed in hexane (ca.15 mg/mL) was first mixed with 5 mL of a dichloromethane solution of $NOBF_4$ (0.01 M) at room temperature. After gently shaking for about 30 min, NPs capped using an ionic $NOBF_4$ ligand were precipitated by the addition of 20 mL mixture of toluene and hexane (1:1 v/v) and then collected by centrifugation at 8000 rpm for 10 min. Subsequently, the $NOBF_4$-capped nanocrystals were re-dispersed in 5 mL of DMF. After adding 150 mg of PAA, the solution was heated to 80 °C for 30 min and then cooled down to room temperature. The PAA-coated NPs were precipitated by the addition of 10 mL of acetone, collected via centrifugation at 6000 rpm for 5 min, washed with ethanol 3 times, and finally dispersed in 3 ml MES solution. The mPEG-NH_2 (4 mg) and 8Arm-PEG-NH_2 (0.8 mg) dissolved in 3 ml MES solution were added. Then, EDC (8 mg) dissolved in MES solution was added, and the solution was shaken for 3 h. The solution was centrifuged at 4000 rpm for 10 min to remove potential large floccules. The

supernate was washed by centrifugal filter (100 kDa) four times to remove excess PEG. The final LnNPs with a cross-linked polymer network were dispersed in 1 x PBS solution at 4 °C for long-term storage.

Conjugation of folic acid on Ln@Y NPs (Ln@Y-FA)

The above Ln@Y NPs with cross-linked polymer network were dispersed in 356 μL 1 x PBS solution (537 μM, Nd@Y-PEG), then 57.4 μL folic acid (0.1 M in DMSO) was added. Subsequently, 86.2 μL of EDC (100 mM) was added drop by drop and the reaction mixture was shaken for 6 h. The solution was centrifuged at 4,000 rpm for 30 min to remove potential large floccules. The supernate was washed by centrifugal filter (100 kDa) twice and then dispersed in 200 μL 1 x PBS solution.

Cell culturing

Mouse 4T1 breast cancer cell lines and L929 fibroblasts cell lines were kindly provided by the Joint Laboratory of Opto-Functional theranostics in Medicine and Chemistry, Jilin University. The selected cell line was cultured in DMEM supplemented with 1% P/S (penicillin and streptomycin）and 10% fetal bovine serum (FBS) (v/v %) at 37 °C under an atmosphere of 5% CO_2.

CCK8 Assay

The in vitro cytotoxicity of ratiometric probes was investigated by the CCK8 method. Briefly, 4T1 cells and L929 cells were respectively seeded in 96-well plates (1×10^4 cells/well) and incubated for 24 h at 37 °C in a humidified incubator with 5% CO_2. Then, the DMEM solution with 0, 18, 37, 73, 146, and 292 mg/mL ratiometric probes (Nd@Y-FA and Er@Y-PEG cocktails) were added and subsequently incubated for 24 h. 10 μL CCK8 solution was carefully added to each well in a 96-well plate, followed by incubation for 4 hours. After incubation, the absorbance value of each well was measured at 450 nm by an Elisa reader (Bio-Tek, Synergy LX, USA) to

evaluate the cytotoxicity of ratiometric probes.

H&E Staining

All the tissues, including the heart, liver, spleen, lung, kidney, tumor, and lymph nodes were fixed in 4% paraformaldehyde after being harvested at 24 h time point post-administration. These tissues were further dehydrated, embedded in paraffin, and sectioned into 3 μm thick slides. H&E staining was then performed according to the protocol of the H&E kit (Beyotime Institute of Biotechnology, Cat. No. C0105). H&E staining images of all tissues were acquired by the upright Nikon Eclipse 80i microscope.

ROC curve

The metastatic status of SLNs was diagnosed by the *in vivo* NIR-II ratiometric fluorescence measurement. Meanwhile, the H&E staining of excised SLNs was performed as the clinical gold standard to verify the accuracy of our method. The quantified results from two methods were processed by prism. The ROC curve was depicted according to the trade-off between the sensitivity and (1-specificity). The cut-off value was calculated with Youden index (sensitivity+specificity-1).

Supplementary Figures

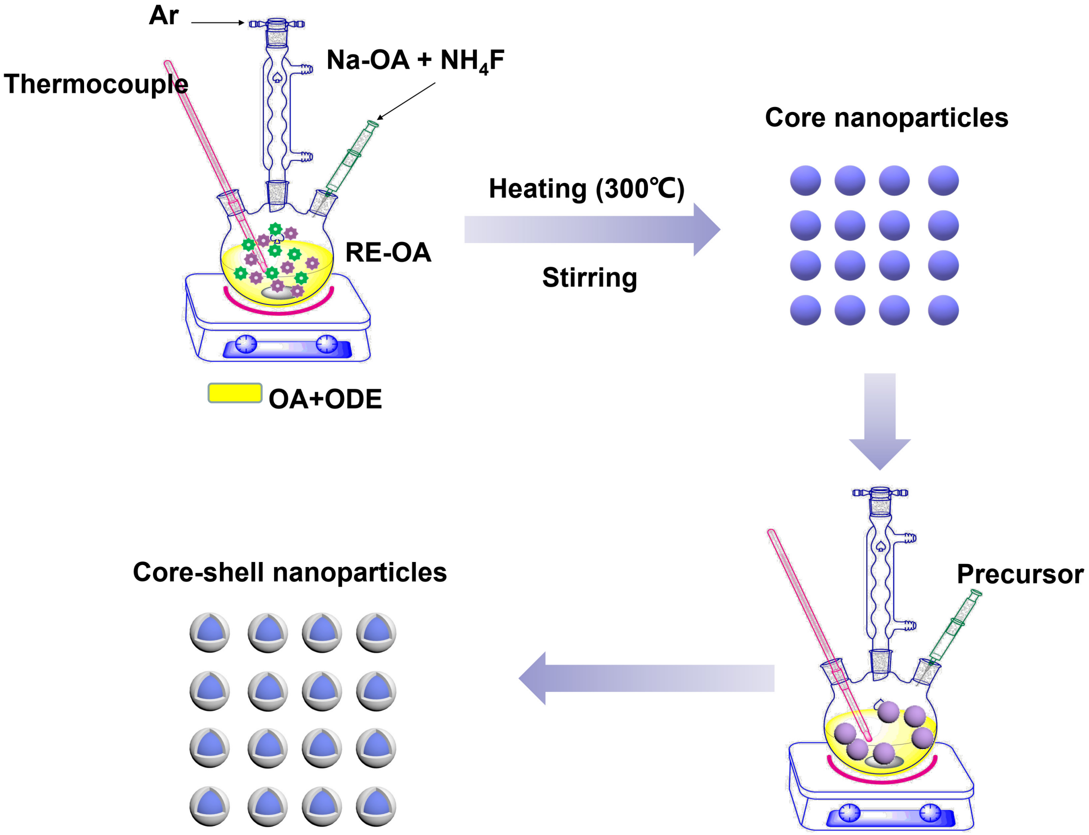

Figure S1. Schematic illustration of the synthesis procedure of core/shell DCNPs. The detailed protocol was listed in the Experimental Section.

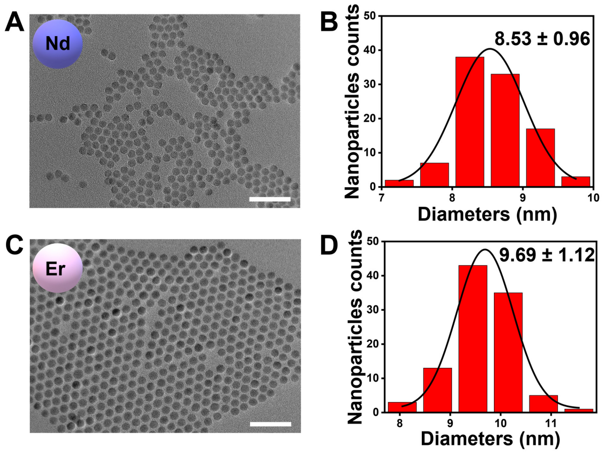

Figure S2. TEM images and histograms of the size distribution for core nanocrystals. (**A, B**) $NaYF_4$:Nd. (**C, D**) $NaErF_4$. All scale bars are 50 nm.

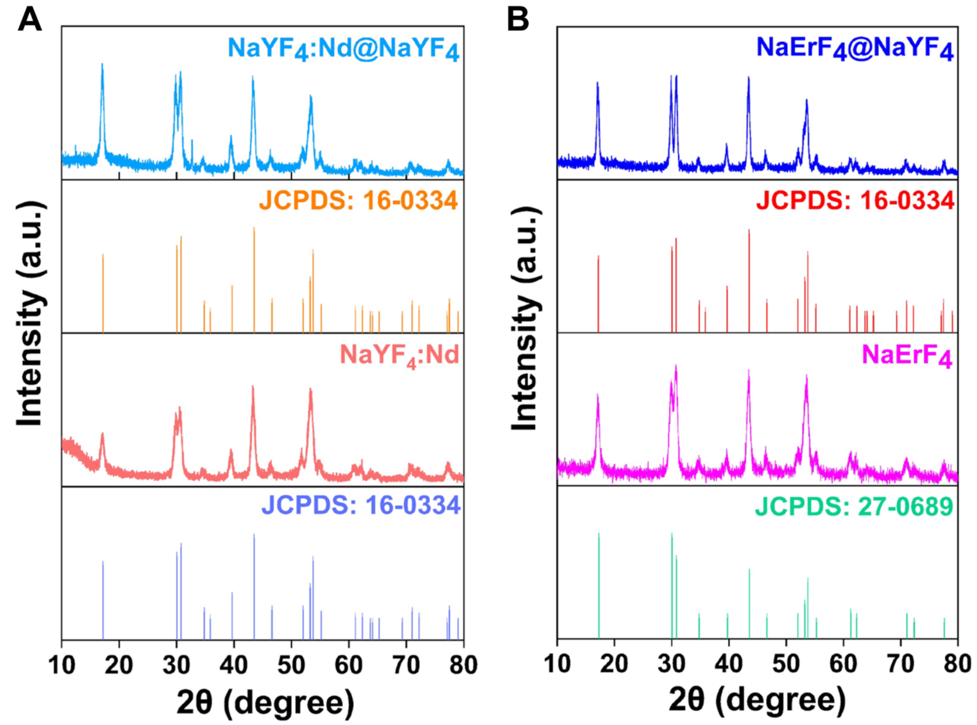

Figure S3. XRD patterns of core and core/shell nanocrystals. (**A**) $NaYF_4$:Nd core, $NaYF_4$:Nd@$NaYF_4$ core@shell, and the standard diffraction patterns of β-NaYF4 (JCPDS 16-0334). (**B**) $NaErF_4$ core, $NaErF_4$@$NaYF_4$ core@shell NPs, and the standard diffraction patterns of β-NaErF4 (JCPDS 27-0689) and β-NaYF4 (JCPDS 16-0334).

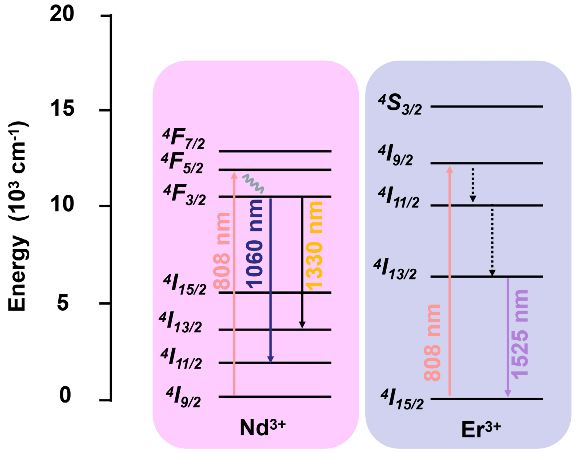

Figure S4. Simplified energy level diagrams of Nd^{3+} and Er^{3+} ions depicting the emitting and energy transfer processes.

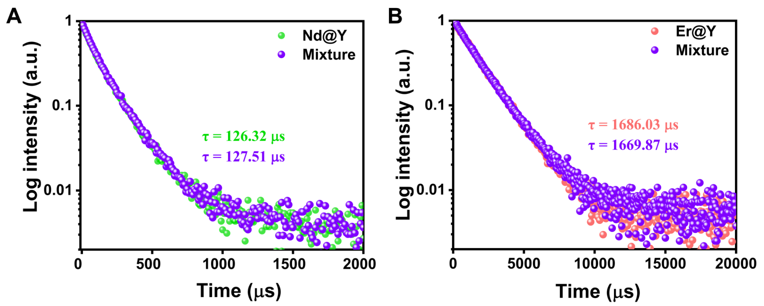

Figure S5. Lifetime characterization of Nd@Y and Er@Y NPs. (**A**) Luminescence decay curves measured at 1060 nm for both Nd@Y NPs and mixture NPs (Nd@Y and Er@Y NPs) under pulsed excitation at 808 nm. (**B**) Luminescence decay curves measured at 1525 nm for both Er@Y NPs and mixture NPs (Nd@Y and Er@Y NPs) under pulsed excitation at 808 nm.

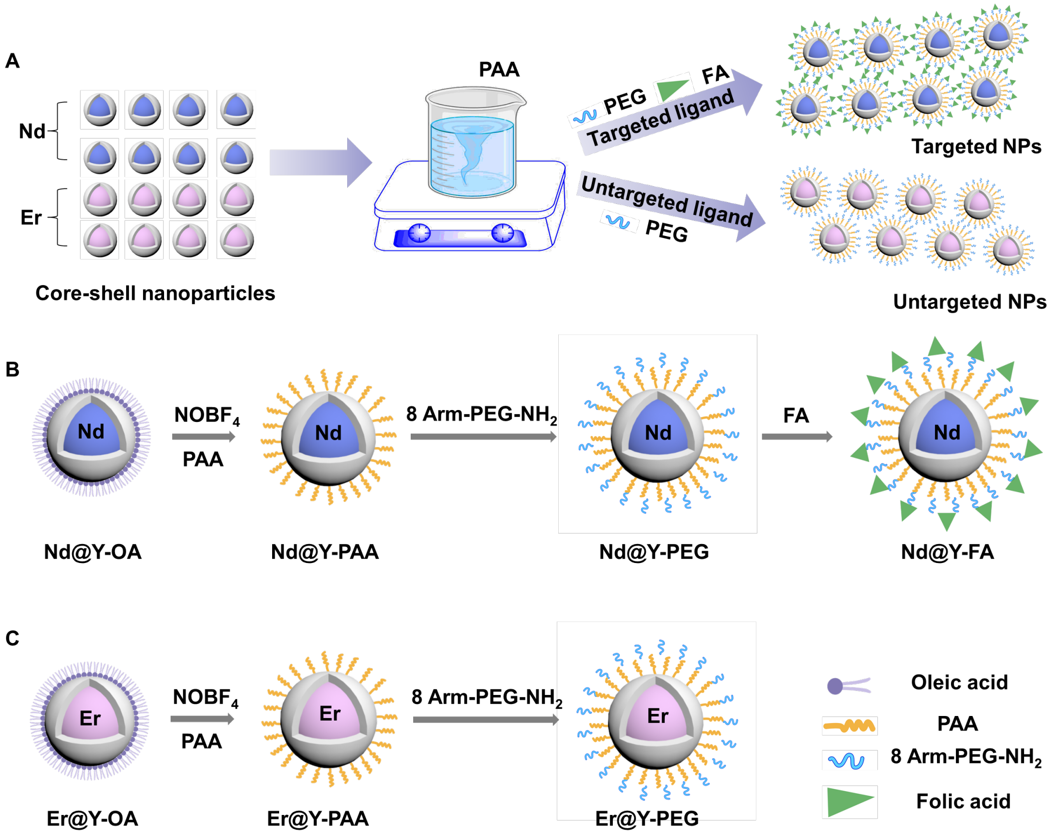

Figure S6. Surface coating for core/shell DCNPs. (**A**) Schematic diagrams showing the surface modification of the NIR-II ratiometric fluorescence probes. Surface modification process of (**B**) targeted probe (Nd@Y-FA) and (**C**) non-targeted probe (Er@Y-PEG).

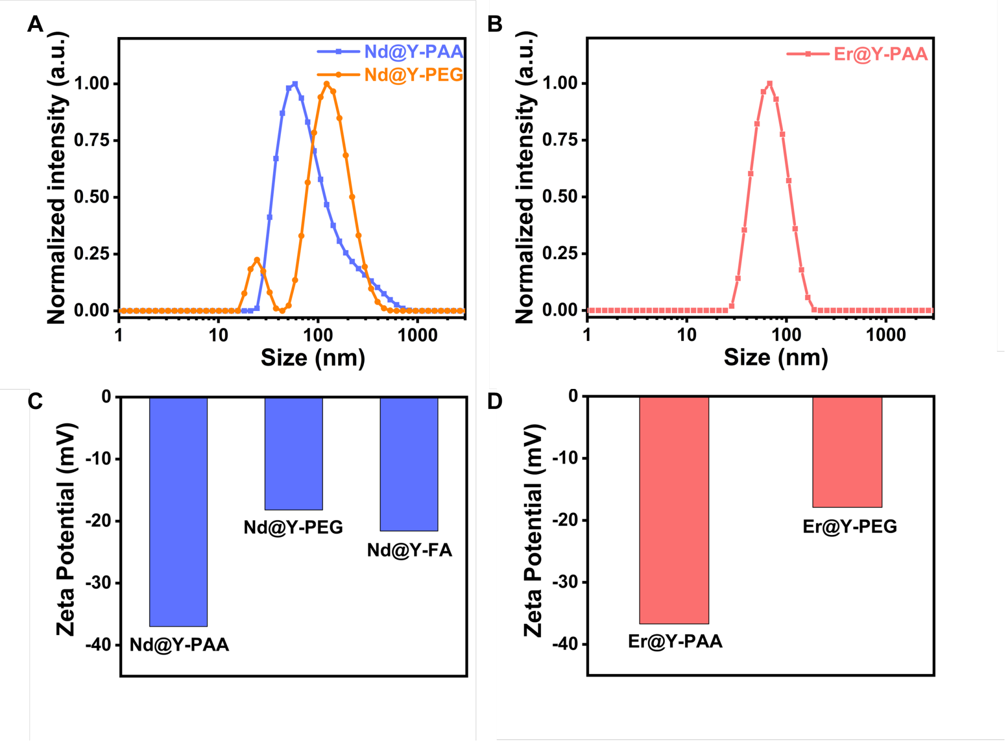

Figure S7. DLS and Zeta potential testing. (**A**) DLS analysis of Nd@Y-PAA NPs, Nd@Y-PEG NPs. (**B**) DLS analysis of Er@Y-PAA NPs. (**C, D**) Zeta potential of all types of NPs.

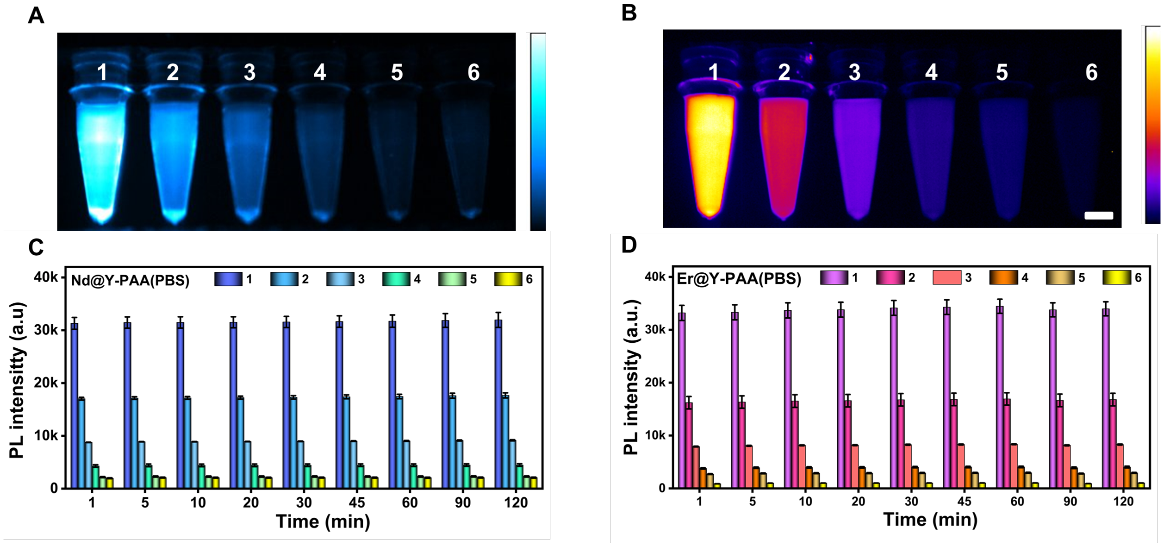

Figure S8. The photo-stability of two PAA-coated NPs under continuous 808 nm laser irradiation. (**A, B**) NIR-II brightness of Nd@Y-PAA NPs and Er@Y-PAA NPs, respectively. The PAA-coated Nd@Y NPs were diluted from 236 to 118, 59, 29.5, 14.8 7.4 mg/mL (1 to 6). The PAA-coated Er@Y NPs were diluted from 306 to 153, 76.5, 38.3, 19.1 9.6 mg/mL (1 to 6). (**C**) The 1060 nm luminescence intensity of Nd@Y-PAA NPs with different concentrations in PBS as a function of irradiation time (2 hours). (**D**) The 1525 nm luminescence intensity of Er@Y-PAA NPs with different concentrations as a function of irradiation time (2 hours). Imaging condition: 808 nm laser excitation with 65 mW/cm^2 power density. All scale bars are 1 cm. We obtained similar results more than three times.

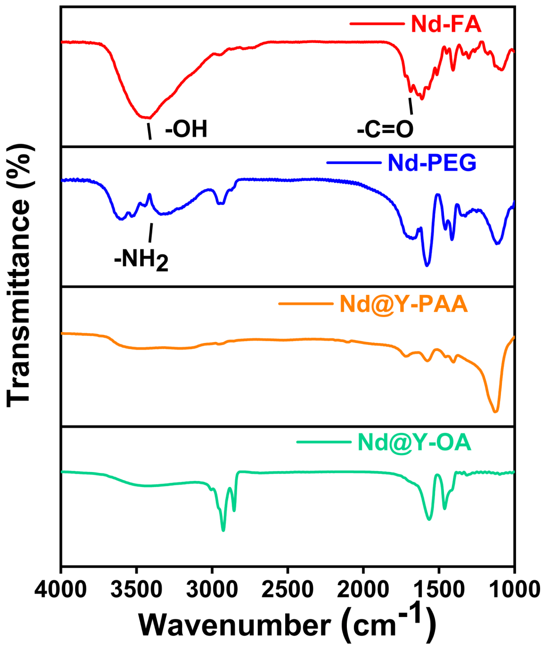

Figure S9. **Fourier transform infrared spectroscopy (FTIR) of Nd@Y-OA NPs, Nd@Y-PAA NPs, Nd@Y-PEG NPs and Nd@Y-FA NPs.**

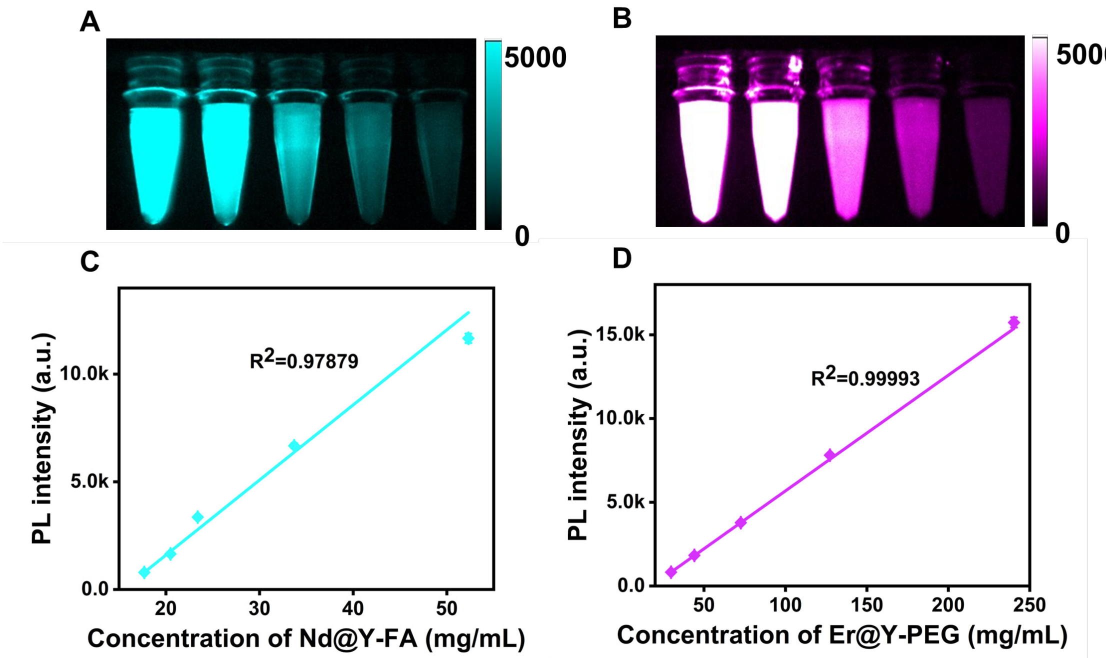

Figure S10**. The linear relationship between the fluorescence intensity and concentration provides an accurate database for quantifying dosage information.** The fluorescence intensity depends linearly on the concentration of ratiometric probes. NIR-II imaging of (**A**) Nd@Y-FA NPs and (**B**) Er@Y-PEG NPs. The solid line represents the linear fitting for (**C**) Nd@Y-FA NPs and (**D**) Er@Y-PEG NPs.

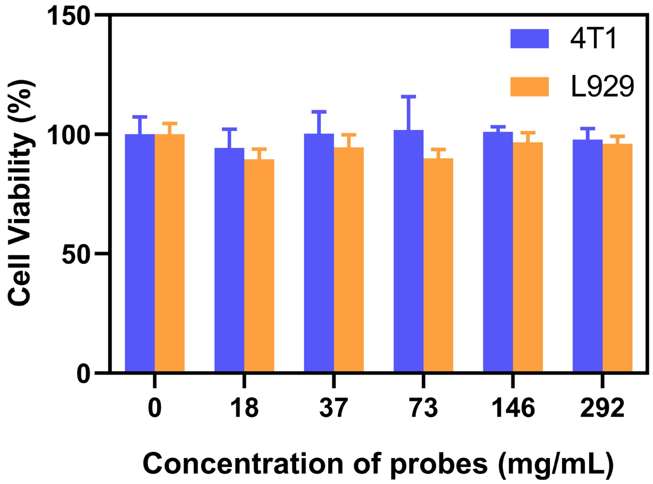

Figure S11. Biosafety of ratiometric probes (Nd@Y-FA and Er@Y-PEG cocktails). The Nd@Y-FA NPs and Er@Y-PEG NPs with equivalent fluorescence intensity under their corresponding emission filters were mixed to form ratiometric probes. Cytotoxicity of ratiometric probes at different concentrations were tested by the breast cancer (4T1) and fibroblasts (L929) cell lines, respectively.

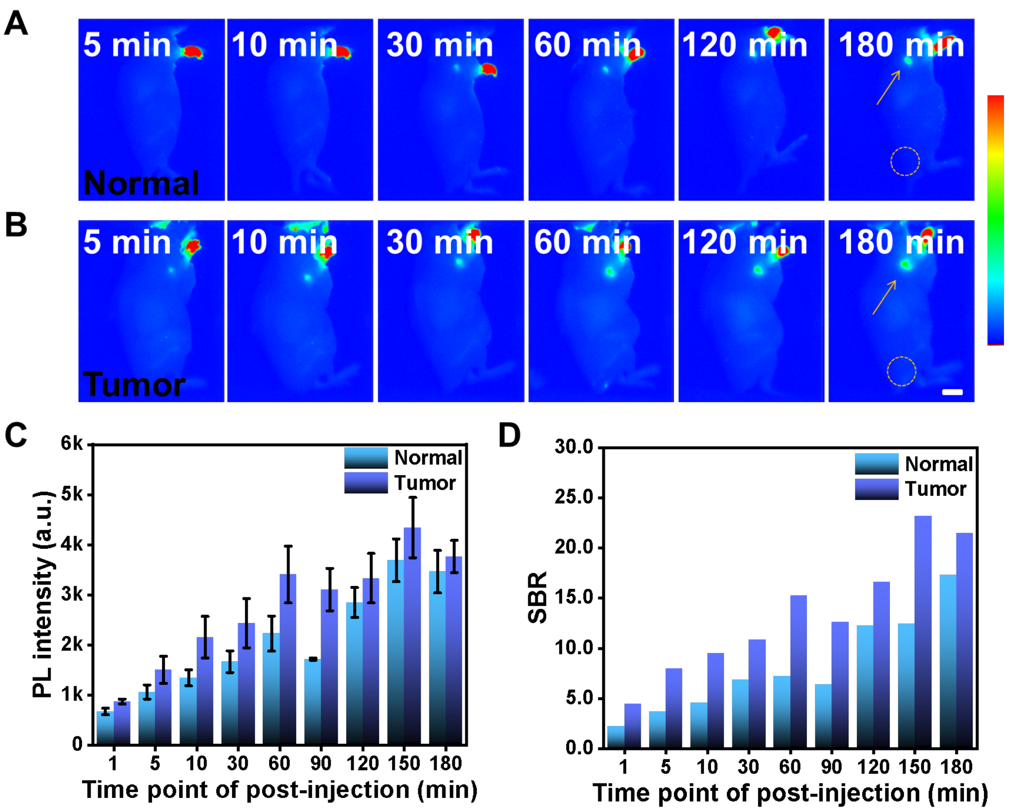

Figure S12. Comparison of SLNs imaging between normal mice and 4T1 tumor-bearing mice with metastatic SLNs by the single targeting probe (Nd@Y-FA). (**A, B**) SLNs were illuminated by Nd@Y-FA probe. (Injection dosage: 62 mg/mL, 25 μL; imaging condition: 808 nm laser excitation with 65 mW/cm^2 power density, 900 and 1000 nm long-pass filters). (**C**) The signals of SLNs were recorded over post-injection time points, indicating the faster migration and more accumulation in tumor mice within 3 hours. (**D**) The signal-to-noise (S/N) ratio was quantified. The brightness of the orange circle area was collected as the background signal.

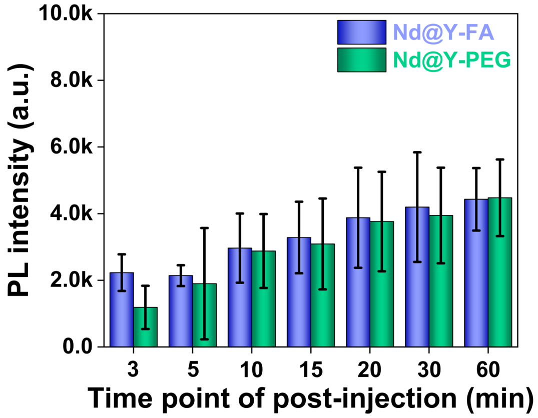

Figure S13. The influence of functional groups FA on the mobility rate of nanoparticles toward SLNs in normal mice. Consistent fluorescence signal variation between Nd@Y-PEG and Nd@Y-FA groups indicated that functional groups FA have moderate influence on the mobility rate of these intradermally injected probes from injected site to the adjacent SLNs. The error bars represented mean ± SD are generated from n = 3 biologically independent mice for each group.

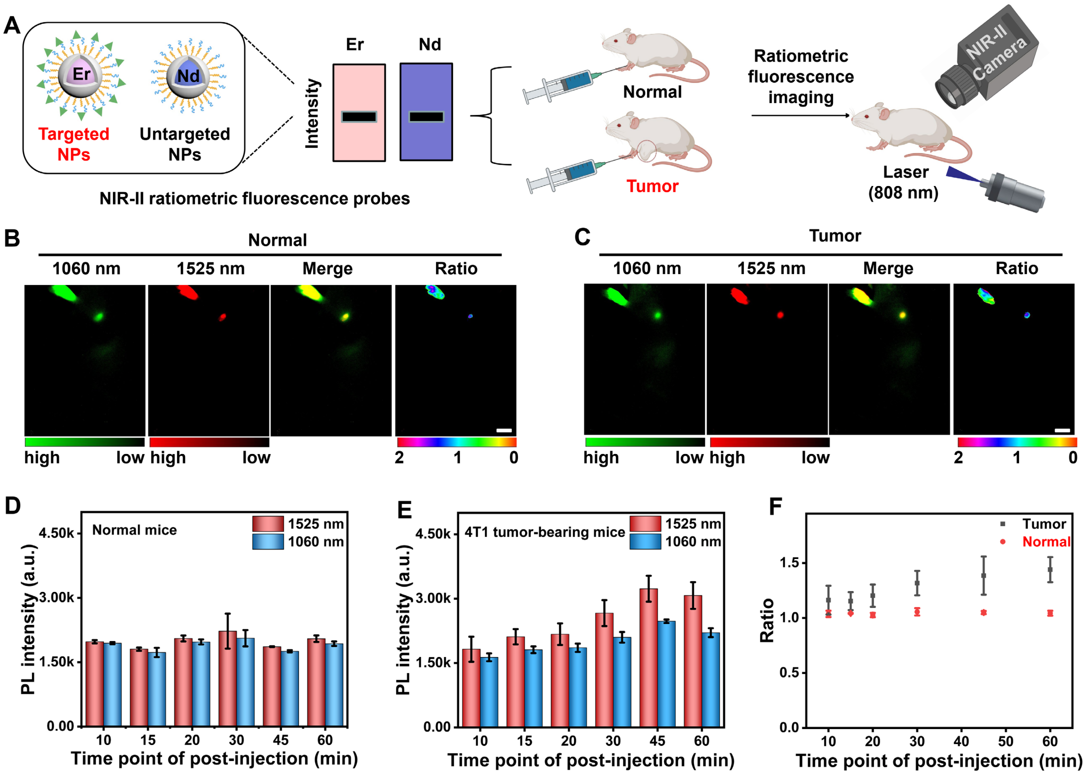

Figure S14. Identification of metastatic SLNs through switching the targeting moiety of two probes to form Er@Y-FA NPs and Nd@Y-PEG NPs pairs. (**A**) Schematic illustration outlining NIR-II ratiometric fluorescence imaging of SLNs in normal and tumor mice using Er@Y-FA NPs and Nd@Y-PEG nanoprobes. Images were created with BioRender.com. (**B, C**) *In vivo* NIR-II fluorescence images with different channels and corresponding merge images of SLNs in normal and tumor metastatic mice excited by a single 808 nm laser. Scale bar: 1 cm. Fluorescence intensities at 1060 nm and 1525 nm of normal mice (**D**) and tumor metastatic mice (**E**) at different post-injection time points. (**F**) NIR-II ratiometric signals of $I_{1525\ nm}/I_{1060\ nm}$ of SLNs were obtained from normal and tumor metastatic groups over time.

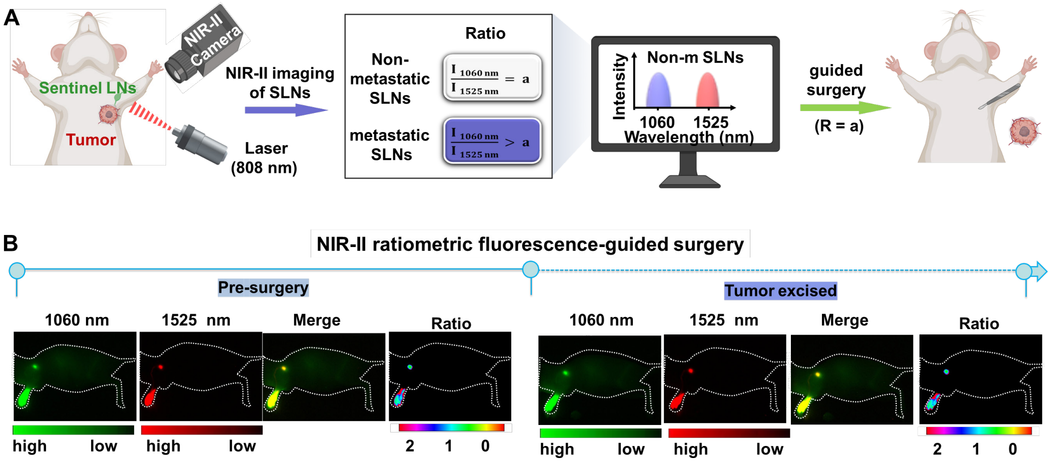

Figure S15. Identification of the metastatic state of SLNs based on NIR-II ratiometric fluorescence. (**A**) Scheme of NIR-II ratiometric fluorescence probes for intraoperative detection and guided surgery in an orthotopic 4T1 breast cancer model. Created with BioRender.com. (**B**) NIR-II ratiometric fluorescence strategy for preoperative diagnosis and intraoperative NIR-II ratiometric-guided surgery in an orthotopic 4T1 breast cancer model.

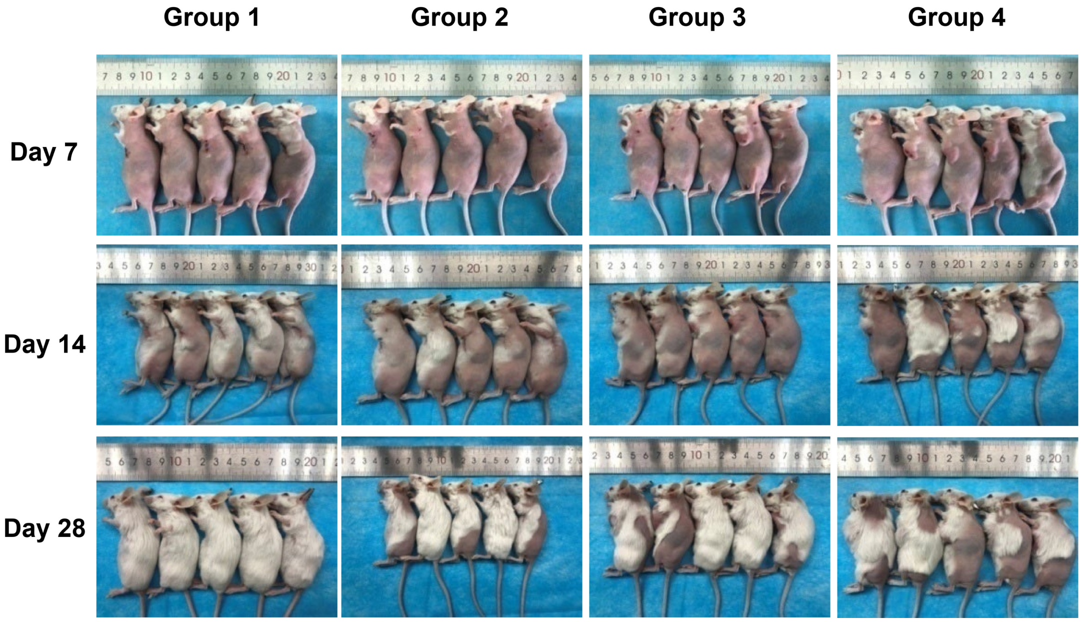

Figure S16. Representative images of Balb/c mice from four cohorts of mice at different time points after surgery. Group 1: The tumor was removed; Group 2: Tumor and SLNs were removed; Group 3: SLNs were removed; Group 4: No treatment as the control.

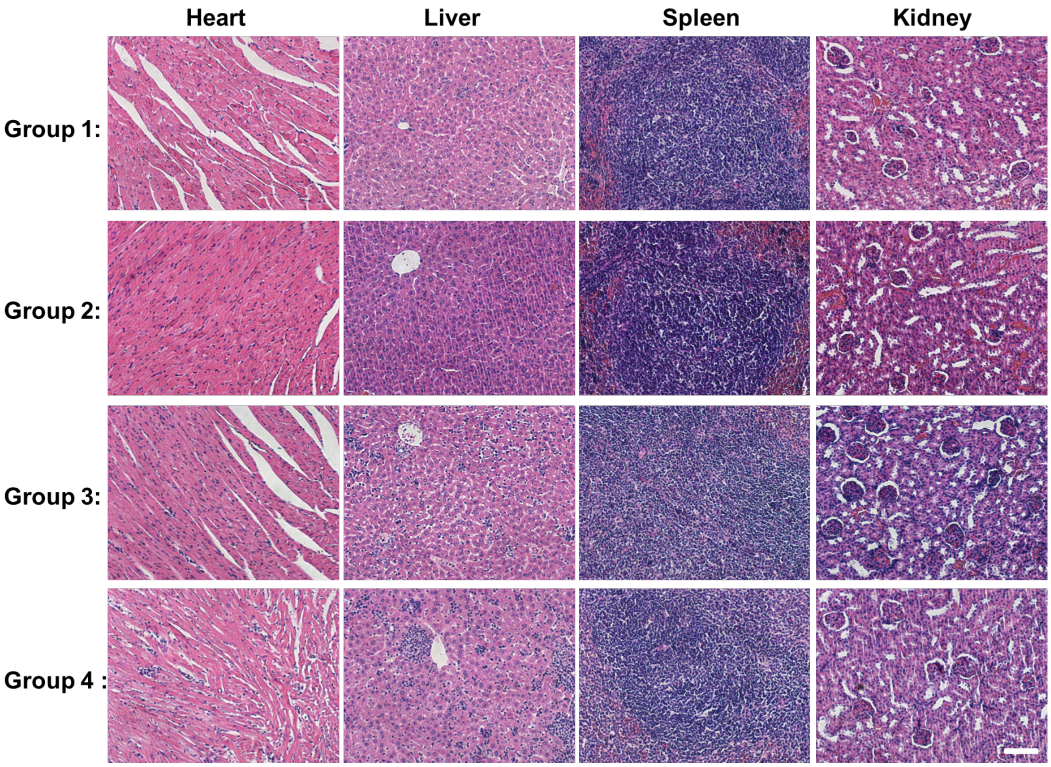

Figure S17. Histological analysis of heart, liver, spleen, and kidneys from all groups. Scale bar: 100 μm for all images.

References

1. Li H, Wang X, Li X, Zeng S, Chen G. Clearable shortwave-infrared-emitting $NaErF_4$ nanoparticles for noninvasive dynamic vascular imaging. Chem Mat. 2020; 32: 3365-75.
2. Zhao L, Kutikov A, Shen J, Duan C, Song J, Han G. Stem cell labeling using polyethylenimine conjugated (α-$NaYbF_4$:Tm^{3+})/CaF_2 upconversion nanoparticles. Theranostics. 2013; 3: 249-57.
3. Zhong Y, Ma Z, Wang F, Wang X, Yang Y, Liu Y, et al. In vivo molecular imaging for immunotherapy using ultra-bright near-infrared-IIb rare-earth nanoparticles. Nat Biotechnol. 2019; 37: 1322-31.
